# Comprehensive evaluation of the metabolic effects of insect meal from *Tenebrio molitor* L. in growing pigs by transcriptomics, metabolomics and lipidomics

**DOI:** 10.1186/s40104-020-0425-7

**Published:** 2020-03-04

**Authors:** Sandra Meyer, Denise K. Gessner, Maria S. Braune, Theresa Friedhoff, Erika Most, Marcus Höring, Gerhard Liebisch, Holger Zorn, Klaus Eder, Robert Ringseis

**Affiliations:** 1 Institute of Animal Nutrition and Nutrition Physiology, Justus Liebig University Giessen, Heinrich-Buff-Ring 26-32, 35392 Giessen, Giessen, Germany; 2 Institute of Clinical Chemistry and Laboratory Medicine, University Hospital of Regensburg, Franz-Josef-Strauss-Allee 11, 93053 Regensburg, Germany; 3 Institute of Food Chemistry and Food Biotechnology, Justus Liebig University Giessen, Heinrich-Buff-Ring 17, 35392 Giessen, Giessen, Germany; 4 Fraunhofer Institute for Molecular Biology and Applied Ecology, Winchester Str. 2, 35394 Giessen, Giessen, Germany

**Keywords:** Animal nutrition, Insect meal, Lipidome, Liver, Metabolism, Metabolome, Pigs, Plasma, Skeletal muscle, Transcriptome

## Abstract

**Background:**

The hypothesis was tested that insect meal (IM) as protein source influences intermediary metabolism of growing pigs. To test this, 30 male, 5-week-old crossbred pigs were randomly assigned to 3 groups of 10 pigs each with similar body weights (BW) and fed isonitrogenous diets either without (CON) or with 5% IM (IM5) or 10% IM (IM10) from *Tenebrio molitor* L. for 4 weeks and key metabolic tissues (liver, muscle, plasma) were analyzed using omics-techniques.

**Results:**

Most performance parameters did not differ across the groups, whereas ileal digestibilities of most amino acids were 6.7 to 15.6%-units lower in IM10 than in CON (*P* < 0.05). Transcriptomics of liver and skeletal muscle revealed a total of 166 and 198, respectively, transcripts differentially expressed between IM10 and CON (*P* < 0.05). Plasma metabolomics revealed higher concentrations of alanine, citrulline, glutamate, proline, serine, tyrosine and valine and a lower concentration of asparagine in IM10 than in CON (*P* < 0.05). Only one out of fourteen quantifiable amino acid metabolites, namely methionine sulfoxide (MetS), in plasma was elevated by 45% and 71% in IM5 and IM10, respectively, compared to CON (*P* < 0.05). Plasma concentrations of both, major carnitine/acylcarnitine species and bile acids were not different across groups. Lipidomics of liver and plasma demonstrated no differences in the concentrations of triacylglycerols, cholesterol and the main phospholipids, lysophospholipids and sphingolipids between groups. The percentages of all individual phosphatidylcholine (PC) and phosphatidylethanolamine (PE) species in the liver showed no differences between groups, except those with 6 double bonds (PC 38:6, PC 40:6, PE 38:6, PE 40:6), which were markedly lower in IM10 than in CON (*P* < 0.05). In line with this, the percentage of C22:6n-3 in hepatic total lipids was lower in IM10 than in the other groups (*P* < 0.05).

**Conclusions:**

Comprehensive analyzes of the transcriptome, lipidome and metabolome of key metabolic tissues indicate that partial or complete replacement of a conventional protein source by IM in the diet has only a weak impact on the intermediary metabolism of growing pigs. Thus, it is concluded that IM from *Tenebrio molitor* L. can be used as a dietary source of protein in pigs without causing adverse effects on metabolism.

## Background

In view of the steadily growing world population and the global trend toward increasing the proportion of animal proteins in the human diet, the global demand for protein as food and feed is increasing. However, it is becoming increasingly difficult to cover this growing demand because of the increasing pressure on limited natural resources such as arable land and water which are required to produce conventional protein sources such as crops and animal proteins. Owing to this, there is an urgent need for alternative protein sources which can be efficiently produced with lower environmental impact [1]. In this regard, protein-rich insect meal (IM) may offer great potential, because it can be produced from industrialized mass-rearing of edible insects (e.g. *Tenebrio molitor* L., *Hermetia illucens*) with higher feed conversion ratios and lower utilization of natural resources than conventional protein sources, at least in theory [2, 3]. Disregarding the legal obstacles currently existing in the EU for the use of IM in farm animal feeding, the use of IM as feed for farm animals in the future presupposes that animals' performance is not impaired in comparison to the use of conventional protein sources and any safety concerns can be excluded. Several studies in birds (broilers, ducks) [4–9] and a few studies in pigs [10–12] already demonstrated that IM as dietary protein source can partially or completely replace soybean extraction meal (SEM) – the major protein source used in diets for monogastric farm animals – without impairing animals' performance. However, studies providing in-depth insights into the effects of IM on intermediary metabolism in farm animals, which are necessary to evaluate potential health risks but also health benefits associated with feeding of IM, are scarce. There are only some largely descriptive studies, in which the effect of a partially defatted IM produced from *Hermetia illucens* has been studied on selected blood parameters (e.g. blood lipids and minerals) and/or histological traits (e.g. histopathological scores) in broilers [7, 8, 13], ducks [14], and pigs [12].

We have recently demonstrated for the first time in two studies with genetically obese rats that IM produced from larvae of the yellow mealworm (*Tenebrio molitor* L.) has remarkable effects on lipid metabolism, such as attenuation of liver steatosis and decrease of hyperlipidemia, − effects which are likely explained by the profound inhibition of hepatic lipid synthesis observed in these studies [15, 16]. Inhibition of hepatic lipid synthesis is possibly mediated by the marked modulation of hepatic phospholipid metabolism [e.g. decrease of hepatic phosphatidylcholine (PC) to phosphatidylethanolamine (PE) ratio] in the obese rats fed the IM [16], because the hepatic PC:PE ratio is a critical determinant of hepatic lipid synthesis [17]. Apart from these modulatory effects on lipid metabolism, largely unexplained effects of IM in the obese rat model were found using screening techniques, such as transcriptomics and metabolomics, on several other metabolic pathways, like metabolism of amino acids and xenobiotics and cellular stress response [15], overall indicating a multifaceted influence of IM on intermediary metabolism of rodents. Despite marked differences exist with regard to intermediary metabolism between rodents and monogastric farm animals and between metabolically impaired (obese) and metabolically healthy (lean) animals, the multifaceted and partially strong metabolic effects observed in obese rats fed IM suggest that IM may also interfere with intermediary metabolism of farm animals. Thus, the present study aimed to test the hypothesis that replacement of SEM by IM influences intermediary metabolism of growing pigs, one of the most important monogastric farm animal species. To test this hypothesis, a feeding trial with weaned piglets receiving isonitrogenous feeding rations, in which SEM was replaced either completely or partially with IM from *Tenebrio molitor* L. larvae, was carried out. In order to investigate the effects of IM on the pigs' metabolism as comprehensively as possible, several omics-techniques such as transcriptomics, metabolomics, and lipidomics were applied on key metabolic tissues, such as liver and skeletal muscle, and plasma of the pigs.

## Methods

### Animals and diets

The four-week feeding trial was approved by the local Animal Care and Use Committee (Regierungspräsidium Giessen; permission no: JLU 676_M). All experimental procedures described followed established guidelines for the care and handling of laboratory animals. The experiment included 30 male, five-week-old crossbred pigs [Piétrain × (German Landrace × German Edelschwein)], which were randomly assigned to three groups of 10 pigs each [Control (CON), 5% IM (IM5), 10% IM (IM10)], with similar initial body weights (BW) (CON: 8.66 ± 1.54 kg; IM5: 8.63 ± 1.49 kg; IM10: 8.79 ± 1.44 kg; mean ± SD; *n* = 10/group). In each treatment group, six pigs were kept in two pens of three animals and four pigs together in one pen under controlled conditions (23 ± 2 °C room temperature, 50-60% relative humidity, light from 07:00 a.m. to 07:00 p.m.). Three diets differing in their main protein source but with comparable levels of gross energy and crude nutrients were fed (Table 1). Nutrient concentrations in the diets were sufficient to meet the requirements according to German Society for Nutrition Physiology [18]. In the diet of group CON, which contained SEM as the main protein source, the amount of IM was 0%, while in the diets of groups IM5 and IM10 the amount of IM was 5% and 10%, respectively, through partial (50%) or complete (100%) isonitrogenous replacement of SEM (44% crude protein) by IM. Owing to the higher crude protein content of the IM (74%) than SEM, cellulose was added in different amounts to the IM5 and IM10 diet. The IM was obtained from *Tenebrio molitor* L. larvae. The analyzed concentrations of crude nutrients, fatty acids and amino acids of the *Tenebrio molitor* L.-IM are shown in Additional file 1: Table S1. *L*-lysine, *DL*-methionine, *L*-threonine, *L*-tryptophan, *L*-glutamic acid and *L*-cysteine were added to the diets in different amounts to adjust amino acid composition of the diets. Owing to the lack of digestibility data of amino acids from the IM used in pigs, amino acid concentrations in the diets are presented as gross concentrations. All diets contained 0.5% titanium dioxide (TiO_2_) which was used to calculate the prececal amino acid digestibility by the indicator method [19]. Owing to the fact that specific fatty acids contained in the IM can cause metabolic effects, the concentrations of the main fatty acids of the three diets were adjusted by addition of individual amounts of rapeseed oil and safflower oil to the diet of group CON and group IM5. The diets were offered ad libitum and feed consumption was recorded daily. BWs were recorded once per week. Water was constantly available *ad libitum* from a nipple drinker system.

**Table 1:** Composition, nutrient and energy contents of the experimental diets

	CON	IM5	IM10
Components, g/kg			
Wheat	380	380	380
Barley	250	250	250
Soybean extraction meal	147.8	73.9	–
Insect meal	–	50	100
Broad bean	110	110	110
Soybean oil	15.0	15.0	15.0
Rapseed oil	4.40	2.10	–
Safflower oil	3.00	1.60	–
Corn starch	3.90	3.00	1.50
Cellulose	–	28.0	56.5
Mineral and vitamin premix^a^	75.0	75.0	75.0
Monocalcium phosphate	6.00	6.00	6.00
Calcium carbonate	0.70	1.10	1.60
*L*-lysine	0.70	1.00	1.40
*DL*-methionine	–	0.10	0.10
*L*-threonine	0.20	0.10	–
*L*-tryptophan	0.10	0.10	0.20
*L*-glutamic acid	3.20	2.60	1.90
*L*-cysteine	–	0.40	0.80
Titanium dioxide	5.00	5.00	5.00
Analyzed crude nutrient and energy content			
Dry matter, % of FM	87.6	87.9	88.5
Crude protein, % of DM	22.2	22.7	22.8
Crude fat, % of DM	4.4	4.3	4.3
Crude ash, % of DM	5.8	5.4	5.0
Crude fiber, % of DM	6.3	8.0	9.7
Gross energy, MJ/kg DM	19.5	19.3	19.6

^a^The mineral and vitamin premix (Bergin TopFit F 75; Bergophor, Kulmbach, Germany) provided per kg diet: 4.35 g, lysin; 1.13 g, methionine; 2.25 g, threonine; 0.19 g, tryptophan; 0.6 g, valine; 15,938 IU, vitamin A; 1875 IU, vitamin D_3_; 150 mg, vitamin E; 120 mg, iron, 150 mg, copper; 112.5 mg, zinc; 75 mg, manganese; 2.25 mg, iodate; 0.45 mg, selenium; 52.5 mg, *L*-carnitine

Abbreviations: *CON* control group, *DM* dry matter, *FM* fresh matter, *IM5* insect meal 5% group; *IM10* insect meal 10% group

### Analysis of feed composition

Determination of total lipid fatty acid composition of the IM, experimental fats and the diets was carried out as described previously [20]. Dry matter content, energy content and concentrations of crude nutrients and amino acids in the diets and the IM were determined by official methods [21, 22]. The chitin content of the insect meal was determined as described recently [16].

### Sample collection

After four weeks, the pigs were killed by exsanguination under electronarcosis in the fed state. Blood was collected into heparin-coated polyethylene tubes (AppliChem, Darmstadt, Germany), and plasma was obtained by centrifugation (1100×*g*; 10 min) at 4 °C. Aliquots of the liver and gastrocnemius muscle were excised, washed in ice-cold NaCl solution (0.9%) and snap-frozen in liquid nitrogen. The gastrointestinal tract was removed and chyme from the ileum was collected. Tissue samples and ileal chyme were snap-frozen in liquid nitrogen and all samples stored at − 80 °C pending analysis.

### RNA extraction

Total RNA from aliquots of liver (20 mg) and gastrocnemius muscle (30 mg) were isolated using TRIzol reagent (Invitrogen, Karlsruhe, Germany) according to the manufacturer’s protocol. RNA quantity and quality were assessed spectrophotometrically using an Infinite 200 M microplate reader equipped with a NanoQuant plate (both from Tecan, Mainz, Germany). The average RNA concentration and the A_260_/A_280_ ratio of all total RNA samples (*n* = 30, means ± SD) were 2.06 ± 0.49 μg/μL and 1.97 ± 0.06 (liver) and 0.54 ± 0.10 μg/μL and 1.96 ± 0.02 (gastrocnemius muscle), respectively.

### Transcript profiling and bioinformatic analysis of microarray data

Transcript profiling in liver and gastrocnemius muscle was carried out for 6 randomly selected pigs per group. Following an RNA quality check using an Agilent 2100 Bioanalyzer (Agilent technologies, Böblingen, Germany), the total RNA samples were processed at the Kompetenzzentrum Fluoreszente Bioanalytik (Regensburg, Germany) using an Affymetrix GeneChip Porcine Gene 1.0 Sense Target array, which represents 19,212 porcine genes, according to the manufacturer’s instructions as described [23]. The average RNA integrity number (RIN) values of all samples (*n* = 18, means ± SD) were 7.91 ± 0.28 (liver) and 7.72 ± 0.41 (gastrocnemius muscle). Following microarray hybridization, analysis of microarray data including background correction and normalization of probe cell intensity data using the Robust Multichip Analysis (RMA) algorithm [24] was carried out as described [23]. The microarray data have been deposited in MIAME compliant format in the NCBI’s Gene Expression Omnibus public repository [25]; GEO accession nos. GSE138241 (skeletal muscle) and GSE138244 (liver)]. Transcripts were defined as differentially expressed when the fold-change (FC) between group IM10 vs. and group CON was > 1.2 or < − 1.2 and the *P*-value of the unpaired Student’s *t*-test (two-tailed distribution, two-sample equal variance) was < 0.05. Similar cut-off FCs have been also used in other animal studies dealing with effects of dietary treatment [23, 26].

Gene set enrichment analysis (GSEA) was performed with the identified differentially expressed transcripts in order to identify enriched Gene Ontology (GO) terms within GO category biological process using the Database for Annotation, Visualization and Integrated Discovery (DAVID) 6.8 bioinformatic resource [27, 28]. GO terms were defined as enriched if *P* < 0.05. GSEA was performed separately for the up- and down-regulated transcripts, respectively.

### Validation of microarray data using qPCR analysis

Microarray data of the top 20 differentially expressed mRNAs were validated by qPCR. For qPCR analysis, total RNA from all pigs (*n* = 10/group) was used. Synthesis of cDNA and qPCR analysis was performed with a Rotor-Gene Q system (Qiagen, Hilden, Germany) as described recently in detail [29]. Gene-specific primers were synthesized by Eurofins MWG Operon (Ebersberg, Germany). Characteristics of primers are listed in Additional file 1: Table S2. Normalization was carried out using multiple reference genes (liver: *GAPDH*, *GPI*, *SDHA*; gastrocnemius muscle: *ATP5MC1*, *GAPDH*, *RPS9*, *SDHA*) as described recently [30]. The mean value calculated from normalized individual values of the control group was set to 1. Mean and SD of group IM5 and group IM10 were scaled proportionally.

### Targeted metabolite screening

Determination of plasma concentrations of specific metabolites from five compound classes [amino acids (*n* = 21), amino acid metabolites (*n* = 21), carnitine species (*n* = 40), bile acids (*n* = 20), sum of hexoses] was carried out using two commercially available kits (Absolute/DQ p180 kit, Bile acids kit) using a flow injection analysis-tandem mass spectrometry (FIA-MS/MS) method for acylcarnitines and hexoses and a liquid chromatography-tandem mass spectrometry (LC-MS/MS) method for amino acids and amino acid metabolites by Biocrates (Innsbruck, Austria).

### Determination of the concentrations of major lipid classes and individual phospholipid species using lipidomics

Lipidomics of plasma and liver was carried out by flow injection analysis (FIA) either using a triple quadrupole instrument (QQQ) or Fourier-transform mass spectrometry (FTMS). The major lipid classes included triacylglycerols (TG), free and esterified cholesterol, phospholipids [phosphatidylcholine (PC), phosphatidylethanolamine (PE), PC-ether (PC O), PE-based plasmalogens (PE P), phosphatidylglycerol (PG), phosphatidylinositol (PI), phosphatidylserine (PS), lysophosphatidylcholine (LPC), lysophosphatidylethanolamine (LPE)] and sphingolipids [ceramide (Cer), hexosylceramide (HexCer), sphingomyelin (SM)]. Lipid extraction was preformed according to the method of Bligh and Dyer [31] in the presence of not naturally occurring lipid species as internal standards. The following lipid species were added as internal standards: PC 14:0/14:0, PC 22:0/22:0, PE 14:0/14:0, PE 20:0/20:0 (di-phytanoyl), PS 14:0/14:0, PS 20:0/20:0 (di-phytanoyl), PI 17:0/17:0, LPC 13:0, LPC 19:0, LPE 13:0, Cer d18:1/14:0, Cer 17:0, D7-FC, CE 17:0 and CE 22:0. Liver homogenates representing a wet weight of 2 mg and 10 μL of serum were extracted. Chloroform phase was recovered by a pipetting robot (Tecan Genesis RSP 150) and vacuum dried. The residues were dissolved in either 10 mmol/L ammonium acetate in methanol/chloroform (3:1, v/v) (for low mass resolution tandem mass spectrometry) or chloroform/methanol/2-propanol (1:2:4 v/v/v) with 7.5 mmol/L ammonium formate (for high resolution mass spectrometry). The analysis of lipids was performed by direct flow injection analysis (FIA) using either a triple quadrupole mass spectrometer (FIA-MS/MS; QQQ triple quadrupole) or a hybrid quadrupole-Orbitrap mass spectrometer (FIA-FTMS; high mass resolution).

FIA-MS/MS (QQQ) was performed in positive ion mode using the analytical setup and strategy described previously [32, 33]. A fragment ion of *m/z* 184 was used for PC, SM [32] and LPC [34]. The following neutral losses were applied: PE and LPE 141, PS 185, PG 189 and PI 277 [35]. PE P were analyzed according to the principles described by Zemski-Berry and Murphy [36]. Sphingosine based Cer d18:1 and HexCer d18:1 were analyzed using a fragment ion of m/z 264 [37]. Lipid species were annotated according to the recently published proposal for shorthand notation of lipid structures that are derived from mass spectrometry [38]. For these data, glycerophospholipid species annotation assumed that only even numbered carbon chains are present. SM species annotation assumed that a sphingoid base with two hydroxyl groups is present.

The Fourier Transform Mass Spectrometry (FIA-FTMS) setup is described in detail in Höring et al. [39]. TG and CE were recorded in positive ion mode FTMS in *m/z* range 500-1000 for 1 min with a maximum injection time (IT) of 200 ms, an automated gain control (AGC) of 1×10^6^, three microscans and a target resolution of 140,000 (at 200 *m/z*). PC O were measured in negative ion mode in *m/z* range 520–960. Multiplexed acquisition (MSX) was used for FC as described [39]. Data processing details were obtained using the ALEX software [40] which includes peak assignment and intensity picking. The extracted data were exported to Microsoft Excel 2010 and further processed by self-programmed Macros.

### Determination of fatty acid composition of hepatic total lipids

Fatty acid composition of hepatic total lipids was determined by gas chromatography-flame ionization detection (GC-FID). Briefly, total lipids were extracted from 50 mg liver aliquots with a 3:2 (v/v)-mixture of n-hexane and isopropanol containing 0.02% butylated hydroxytoluene. After extraction, samples were centrifuged (1200×*g*, 10 min) and an aliquot of the supernatant was evaporated under a stream of N_2_ at 37 °C. Lipids were subsequently transmethylated using trimethylsulfonium hydroxide solution (Sigma-Aldrich) [41] and the resulting fatty acid methyl esters (FAMEs) were separated by a GC-FID system described in detail recently [30].

### Determination of the concentrations of amino acids and TiO_2_ in ileal chyme and calculation of ileal digestibilities of amino acids

Prior to analysis, ileal samples were freeze-dried and ground using a centrifugal mill (Retsch, Haan, Germany). The concentration of the indigestible indicator TiO_2_ in the ileal chyme was determined by the method of Brandt and Allam with slight modifications [42]. Tryptophan in the ileal chyme was measured according to a modified version of the official methods [22]. Briefly, 100 mg aliquots of the chyme were incubated in barium hydroxide solution in an ultrasonic bath for 15 min, and then incubated at 110 °C for 24 h under nitrogen atmosphere. Afterwards, distilled water was added and samples were cooled on ice. Following addition of ortho-phosphoric acid, 6 mol/L hydrochloric acid (HCl) and internal standard, the pH was adjusted to 3.0 and samples were diluted 1:20 fold with distilled water. After a centrifugation step (20,000×*g*, 10 min, 10 °C), the supernatant was measured by reversed phase-high performance liquid chromatography with fluorescence detection (excitation wavelength: 283 nm, emission wavelength: 355 nm). The remaining amino acids were measured following acidic hydrolysis with HCl according to the official method [22]. In brief, ileal chyme aliquots (10-20 mg) were weighed in hydrolysis tubes and 6 mol/L HCl was added to the tubes. Subsequently, samples were deep-frozen at − 62 °C, merged under vacuum and hydrolyzed at 115 °C for 24 h. Afterwards, samples were dried at 36 °C for 8 h in a vacuum centrifuge, dissolved in sodium-acetate (pH 2.2) and centrifuged. An aliquot from the clear supernatant was analyzed using an amino acid analyzer (LC 3000, Biotronik, Berlin, Germany) by separation on a polymer cation exchanger column (particle size 4 μm, internal diameter 125 × 4 mm) with post-column derivatization at 125 °C using ninhydrin and photometric detection at 570 nm. For the determination of methionine and cysteine, ileal chyme was subjected to oxidation with freshly prepared performic acid at 4 °C for 24 h to generate hydrolysis-stable derivates prior to acidic hydrolysis. Based on the dietary and ileal chyme concentrations of indicator and amino acids, respectively, the ileal digestibility was calculated by the indicator method according to Yin et al. [19].

### Statistical analysis

Statistical analyses were performed using the Minitab statistical software (Rel. 13.1, Minitab, State College, PA, USA). The experimental unit was the pen for performance data and the individual animal for all other data. Performance data were subjected to 2-factorial ANOVA with classification factors being diet, stocking density and the interaction of both factors. All other data were checked for distribution of normality of the residuals by Anderson-Darling test. Normally distributed data were analyzed by one-way ANOVA followed by Fisher’s multiple comparison test. If data were not normally distributed, the non-parametric Kruskal-Wallis test was used for inter-group comparison and the Bonferroni-corrected Mann-Whitney *U* test for between-group comparison. Differences were considered significant at *P* < 0.05. Correlation analysis was carried out with the linear regression tool from Minitab. Correlation was considered significant at *P* < 0.05.

## Results

### Characterization of the experimental diets

The concentrations of crude protein and gross energy did not differ across the three diets indicating that partial (IM5 diet) and complete (IM10 diet) replacement of SEM by IM was isonitrogenous and isocaloric. While the concentration of crude fat was similar across the three diets, the crude ash concentration was 14% lower in the IM10 diet than in the CON diet. Due to addition of cellulose to the IM5 and IM10 diet, the concentration of crude fiber was 27% and 54% higher in the IM5 and the IM10 diet, respectively, than in the CON diet. Despite the use of different amounts of the main protein sources (SEM, IM), the concentrations of amino acids in the diets were relatively similar between the three diets owing to the supplementation of individual amounts of *L*-lysine, *DL*-methionine, *L*-threonine, *L*-tryptophan, *L*-glutamic acid and *L*-cysteine (Table 2). The concentrations of crude fat and major fatty acids (linoleic acid, oleic acid, palmitic acid, stearic acid and α-linolenic acid), which accounted for more than 95% of total fatty acids, were similar in the three diets owing to the adjustment of fat content and fatty acid composition by addition of individual amounts of rapeseed oil and safflower oil (Table 2).

**Table 2:** Concentrations of amino acids and total lipid fatty acid composition in the experimental diets

	CON	IM5	IM10
Amino acids, g/kg FM			
Alanine	6.7	7.8	8.4
Arginine	10.8	10.3	9.8
Aspartic acid	12.6	12.4	11.3
Cysteine	4.8	5.0	5.0
Glutamic acid	40.0	39.7	35.9
Glycine	7.0	7.4	7.6
Histidine	5.0	5.2	5.3
Isoleucine	7.4	7.7	7.6
Leucine	12.1	12.6	12.4
Lysine	11.8	13.6	13.4
Methionine	2.6	2.7	2.7
Phenylalanine	7.9	7.9	7.3
Proline	10.5	11.6	12.1
Serine	6.8	6.9	6.7
Taurine	3.2	3.2	3.2
Threonine	6.8	8.0	7.6
Tryptophan	2.5	2.5	2.6
Tyrosine	5.5	6.4	6.7
Valine	8.6	9.3	9.8
Fatty acids^1^, g/100 g total FAME			
12:0	0.3	0.3	0.6
14:0	0.6	0.5	0.7
16:0	14.6	16.0	16.7
16:1n-9	–	0.3	0.8
18:0	5.0	4.7	5.0
18:1n-9	27.3	25.7	25.2
18:2n-6	48.2	48.3	47.3
18:3n-3	3.6	3.8	3.4
20:0	0.3	0.3	0.3

^1^Only fatty acids with concentrations ≥0.1 g/100 g total fatty acids are shown

Abbreviations: *CON* control group; *FAME* fatty acid methyl esters; *FM* fresh matter; *IM5* insect meal 5% group; *IM10* insect meal 10% group

### Effect on the growth performance and ileal digestibilities of amino acids of the pigs

Most performance parameters (daily feed intake, final BW, gain:feed-ratio) did not differ across the three groups (Table 3). Only daily BW gain was lower in group IM10 compared to group IM5 and group CON (*P* < 0.05; Table 3). BW gain was affected by diet, stocking density and the interaction of both factors.

**Table 3:** Performance parameters of pigs fed isonitrogenous diets without (CON) or with 5% insect meal (IM5) or 10% insect meal (IM10) for 4 weeks

				2-way ANOVA		
	CON	IM5	IM10	Diet	Stocking density	Diet × Stocking density
Initial BW, kg	8.66 ± 1.54	8.63 ± 1.49	8.79 ± 1.44	0.983	0.150	0.343
Final BW, kg	26.5 ± 3.8	25.8 ± 3.1	24.8 ± 3.0	0.100	0.065	0.067
Daily BW gain, g	636 ± 91	614 ± 60	573 ± 67	0.003	0.037	0.009
Daily feed intake, g	868 ± 107	866 ± 47	860 ± 91	0.581	0.476	0.127
Gain:feed-ratio, g/kg	734 ± 56	704 ± 33	670 ± 46	0.558	0.960	0.259

Values are means ± SDs for *n* = 3 pens per group

Abbreviations: *BW* body weight; *CON* control group; *FM* fresh matter; *IM5* insect meal 5% group; *IM10* insect meal 10% group

Ileal digestibility of all amino acids, except aspartic acid, were 6.7 to 15.6%-units lower in pigs of group IM10 than in pigs of group CON (Additional file 1: Table S3). The ileal digestibility of aspartic acid was 26.3%-units lower in group IM10 than in group CON.

### Effect on the hepatic transcriptome of the pigs

A total of 166 transcripts (105 up-regulated, 61 down-regulated) were found to be differentially expressed in the liver between group IM10 and group CON according to the filter criteria applied (FC > 1.2 or < − 1.2 and *P* < 0.05). All transcripts including FCs and *P*-values are shown in Additional file 1: Table S4. The most strongly up-regulated transcript was retinol dehydrogenase 16 (*LOC100512656*; FC = 4.45), while the most strongly down-regulated transcript was transmembrane protein 52B (*TMEM52B*; FC = − 3.44). In Additional file 1: Table S4, the FCs and *P*-values are also presented for the same transcripts for the comparison between group IM5 and group CON. According to this, 88% from the 105 up-regulated transcripts and 92% from the 61 down-regulated transcripts were less strong regulated between group IM5 vs. CON than between group IM10 vs. CON. Microarray data were validated by qPCR analysis of the 20 most strongly regulated mRNAs. When comparing the FCs of the mRNAs to be validated between microarray and qPCR data, the effect direction was the same in 19 cases (Additional file 1: Table S5). One mRNA (*CUEDC*) which was found to be upregulated according to microarray analysis, was almost not regulated according to qPCR data. Statistical analysis of qPCR data showed that 11 out of the 20 mRNAs to be validated were differentially regulated between group IM10 and group CON at a significance level of *P* < 0.05.

To identify biological processes affected by the differentially regulated transcripts, GSEA was performed using GO biological process terms. GSEA of the up-regulated transcripts revealed the following as the most enriched GO biological process terms (first lowest *P*-value, last highest *P*-value): regulation of sequence-specific DNA binding transcription factor activity, negative regulation of transmembrane receptor protein serine/threonine kinase signaling pathway, DNA-templated transcription, tube development, rhythmic process, leukocyte activation involved in immune response, cell activation involved in immune response, cell development, negative regulation of sequence-specific DNA binding transcription factor activity, and BMP signaling pathway. The whole list of biological process terms with *P*-values < 0.05 including the number of genes assigned to these terms is shown in Table 4. GSEA of the down-regulated mRNAs demonstrated that the most enriched biological process terms were related to urea metabolic process, urea cycle, nitrogen cycle metabolic process, cellular response to chemical stimulus, arginine metabolic process, negative regulation of establishment of protein localization, leukocyte cell-cell adhesion, and protein folding. Table 4 shows the GO biological process terms with *P*-values < 0.05 including the number of genes assigned to these terms.

**Table 4:** The most enriched GO biological process terms assigned to the 105 up-regulated and 61 down-regulated transcripts in the liver of pigs of group IM10 compared with group CON^a^

GO biological process term	Number of genes	*P*-value	Fold enrichment
Up-regulated transcripts			
Regulation of sequence-specific DNA binding transcription factor activity	7	0.001	5.8
Negative regulation of transmembrane receptor protein serine/threonine kinase signaling pathway	4	0.004	12.6
Transcription, DNA-templated	18	0.005	2.0
Tube development	8	0.005	3.7
Rhythmic process	5	0.007	6.4
Leukocyte activation involved in immune response	5	0.010	5.8
Cell activation involved in immune response	5	0.010	5.8
Cell development	14	0.011	2.1
Negative regulation of sequence-specific DNA binding transcription factor activity	4	0.011	8.5
BMP signaling pathway	4	0.014	7.8
Cellular process involved in reproduction in multicellular organism	5	0.016	5.1
Response to BMP	4	0.016	7.4
Cellular response to BMP stimulus	4	0.016	7.4
Termination of RNA polymerase I transcription	2	0.017	115.2
Regulation of gene expression	20	0.021	1.7
Regulation of macromolecule biosynthetic process	19	0.021	1.7
Transmembrane receptor protein serine/threonine kinase signaling pathway	5	0.021	4.7
Regulation of cell differentiation	11	0.022	2.2
Nucleic acid-templated transcription	18	0.022	1.7
Positive regulation of macromolecule metabolic process	16	0.023	1.8
Positive regulation of cellular metabolic process	16	0.023	1.8
RNA biosynthetic process	18	0.024	1.7
Transcription from RNA polymerase II promoter	11	0.027	2.2
Fc receptor signaling pathway	2	0.028	69.1
Cellular response to chemical stimulus	14	0.032	1.8
Regulation of cellular macromolecule biosynthetic process	18	0.033	1.6
Lipid metabolic process	9	0.035	2.3
Regulation of transmembrane receptor protein serine/threonine kinase signaling pathway	4	0.037	5.4
Nucleobase-containing compound biosynthetic process	19	0.037	1.6
Positive regulation of metabolic process	16	0.039	1.7
Regulation of transcription from RNA polymerase II promoter	11	0.042	2.0
Regulation of nitrogen compound metabolic process	19	0.043	1.6
Heterocycle biosynthetic process	19	0.044	1.6
Positive regulation of macromolecule biosynthetic process	10	0.044	2.1
Negative regulation of transport	5	0.045	3.7
Positive regulation of kinase activity	5	0.045	3.7
Aromatic compound biosynthetic process	19	0.045	1.6
Regulation of osteoblast differentiation	3	0.046	8.6
Negative regulation of cell differentiation	6	0.048	3.0
Endothelial cell differentiation	3	0.049	8.4
Positive regulation of protein serine/threonine kinase activity	4	0.049	4.8
Down-regulated transcripts			
Urea metabolic process	2	0.021	91.0
Urea cycle	2	0.021	91.0
Nitrogen cycle metabolic process	2	0.026	74.5
Cellular response to chemical stimulus	8	0.028	2.5
Arginine metabolic process	2	0.032	58.5
Negative regulation of establishment of protein localization	3	0.033	10.2
Leukocyte cell-cell adhesion	4	0.034	5.4
Protein folding	3	0.039	9.2

^a^GO terms are sorted by their fold enrichment score in descending order. Only GO terms with EASE scores (enrichment *P*-value) < 0.05 are shown

Abbreviations: *CON* control group; *GO* gene ontology; *IM10* insect meal 10% group

### Effect on the skeletal muscle’s transcriptome of the pigs

In gastrocnemius muscle, 198 transcripts (87 up-regulated, 111 down-regulated) were identified as differentially expressed (FC > 1.2 or < − 1.2 and *P* < 0.05) between group IM10 and group CON (Additional file 1: Table S6). Like in the liver, the most strongly up-regulated transcript in group IM10 was retinol dehydrogenase 16 (*LOC100512656*; FC = 5.09). The most strongly down-regulated transcript was BTG family, member 2 (*BTG2*; FC = − 3.63). Additional file 1: Table S6 also contains the FCs and *P*-values for the same transcripts for the comparison between group IM5 and group CON. This comparison showed that 87% from the 87 up-regulated transcripts and 92% from the 111 down-regulated transcripts were regulated less strong between group IM5 vs. CON than between group IM10 vs. CON. Validation of microarray data for 20 differentially regulated mRNAs by means of qPCR analysis revealed the same effect direction in most cases except *GBE1*, which was not regulated according to qPCR (Additional file 1: Table S7). Statistical analysis of qPCR data demonstrated significant differential regulation (*P* < 0.05) between group IM10 and group CON only for 8 of the mRNAs to be validated (*LOC100512656*, *ABRA*, *ATF5*, *OLFM3*, *BTG2*, *ARG2*, *ENHO*, *IFRD1*).

Identification of biological processes affected by the differentially regulated transcripts was carried out by GSEA. GSEA of the up-regulated transcripts revealed only the following three enriched GO biological process terms (*P* < 0.05): G-protein coupled receptor signaling pathway, phagocytosis and glucose 6-phosphate metabolic process. The exact *P*-values and the number of genes assigned to these terms are shown in Table 5. In the case of the down-regulated mRNAs, GSEA revealed more enriched biological process terms, with the lowest *P*-values being: response to lipid, neurological system process, adult behavior, neuron development, system process, response to retinoic acid, and neuron projection development. All GO biological process terms with *P*-values < 0.05 and the number of genes assigned to these terms are presented in Table 5.

**Table 5:** The most enriched GO biological process terms assigned to the 87 up-regulated and 111 down-regulated transcripts in gastrocnemius muscle of pigs of group IM10 compared with group CON^a^

GO biological process term	Number of genes	*P*-value	Fold enrichment
Up-regulated transcripts			
G-protein coupled receptor signaling pathway	11	0.004	2.8
Phagocytosis	3	0.034	10.1
Glucose 6-phosphate metabolic process	2	0.036	54.0
Down-regulated transcripts			
Response to lipid	7	0.009	3.8
Neurological system process	11	0.011	2.5
Adult behavior	4	0.013	8.0
Neuron development	8	0.019	2.9
System process	13	0.020	2.0
Response to retinoic acid	3	0.021	13.2
Neuron projection development	7	0.026	3.0
Single-organism behavior	5	0.026	4.3
Adult locomotory behavior	3	0.037	9.7
Sensory perception of chemical stimulus	7	0.038	2.8
Axon development	5	0.039	3.8
Sensory perception of smell	5	0.041	3.8
Response to organic substance	13	0.042	1.8
Kidney development	4	0.049	4.8

^a^GO terms are sorted by their enrichment *P*-value in ascending order. Only GO terms with EASE scores (enrichment *P*-value) < 0.05 are shown

Abbreviations: *CON* control group; *GO* gene ontology; *IM10* insect meal 10% group

### Effect on plasma amino acids, amino acid metabolites, carnitine species and bile acids of the pigs

The plasma concentrations of alanine, citrulline, glutamate, proline, serine, tyrosine and valine were 19%, 15%, 33%, 27%, 19%, 46% and 62%, respectively, higher in group IM10 than in group CON, whereas the concentration of asparagine was 24% lower in group IM10 than in group CON (*P* < 0.05; Table 6). The plasma concentrations of proline, tyrosine and valine were also 16%, 25% and 34%, respectively, higher in group IM5 than in group CON. Plasma concentrations of all other amino acids did not differ across the groups. Plasma concentrations of all quantifiable amino acid metabolites (*n* = 14) were not different between the three groups, except methionine sulfoxide (MetS), which was markedly elevated by 45% and 71% in group IM5 and group IM10, respectively, compared to group CON (*P* < 0.05; Table 6).

**Table 6:** Plasma concentrations of amino acids and amino acid metabolites of pigs fed isonitrogenous diets without (CON) or with 5% insect meal (IM5) or 10% insect meal (IM10) for 4 weeks

	CON	IM5	IM10	*P*-value
Amino acids, μmol/L				
Alanine	725 ± 74^b^	720 ± 95^b^	860 ± 88^a^	0.002
Arginine	191 ± 32	191 ± 39	175 ± 40	0.532
Asparagine	101.3 ± 19.4^a^	89.2 ± 18.3^ab^	77.1 ± 14.3^b^	0.017
Aspartate	27.8 ± 4.5	27.0 ± 8.2	29.4 ± 5.7	0.704
Citrulline	80.4 ± 14.6^b^	78.8 ± 8.7^b^	92.8 ± 12.9^a^	0.038
Glutamine	775 ± 86	741 ± 129	746 ± 104	0.747
Glutamate	344 ± 36^b^	374 ± 138^ab^	457 ± 92^a^	0.041
Glycine	1267 ± 148	1401 ± 235	1421 ± 252	0.241
Histidine	37.6 ± 11.9	43.0 ± 11.4	38.1 ± 9.1	0.485
Isoleucine	256 ± 51	241 ± 44	242 ± 30	0.693
Leucine	180 ± 48	213 ± 36	202 ± 39	0.219
Lysine	499 ± 47	545 ± 141	493 ± 109	0.500
Methionine	66.9 ± 16.4	78.6 ± 20.5	65.6 ± 19.8	0.039
Ornithine	140 ± 16	148 ± 41	139 ± 28	0.771
Phenylalanine	81.4 ± 12.4	75.7 ± 9.6	69.2 ± 13.6	0.094
Proline	485 ± 73^b^	561 ± 72^a^	618 ± 62^a^	0.001
Serine	172 ± 26^b^	187 ± 26^ab^	204 ± 23^a^	0.035
Threonine	348 ± 78	402 ± 84	388 ± 134	0.472
Tryptophan	82.3 ± 10.3	73.8 ± 11.1	74.8 ± 13.6	0.234
Tyrosine	129 ± 24^c^	161 ± 20^b^	188 ± 24^a^	0.001
Valine	393 ± 69^c^	526 ± 60^b^	635 ± 84^a^	0.001
Amino acid metabolites, μmol*/L*				
Asymmetric dimethylarginine	1.85 ± 0.19	1.89 ± 0.18	1.94 ± 0.17	0.524
Alpha-aminoadipic acid	49.5 ± 10.5	47.7 ± 13.3	40.1 ± 18.2	0.316
Carnosine	17.4 ± 2.6	16.7 ± 3.8	15.8 ± 3.4	0.568
Creatinine	70.3 ± 8.2	67.8 ± 8.1	67.4 ± 9.9	0.728
Histamine	1.28 ± 1.17	1.01 ± 0.80	1.31 ± 0.94	0.806
Kynurenine	0.99 ± 0.17	0.93 ± 0.12	1.00 ± 0.21	0.691
Methionine sulfoxide	3.28 ± 0.78^b^	4.77 ± 1.24^a^	5.61 ± 1.44^a^	0.001
Putrescine	1.10 ± 0.17	1.11 ± 0.24	1.17 ± 0.14	0.628
Sarcosine	2.23 ± 0.34	2.41 ± 0.35	2.63 ± 0.56	0.140
Symmetric dimethylarginine	0.85 ± 0.11	0.84 ± 0.09	0.92 ± 0.09	0.084
Serotonin	0.39 ± 0.40	0.46 ± 0.38	0.47 ± 0.41	0.705
Spermidine	0.83 ± 0.35	0.80 ± 0.37	0.68 ± 0.35	0.555
Trans-4-hydroxyproline	115 ± 16	123 ± 14	122 ± 7	0.328
Taurine	64.3 ± 14.9	74.7 ± 20.0	69.9 ± 16.2	0.418

Values are means ± SDs for *n* = 10 pigs per group. Means without a common superscript letter differ, *P* < 0.05

Abbreviations: *CON* control group; *IM5* insect meal 5% group; *IM10* insect meal 10% group

Out of 40 plasma carnitine species detectable, only seven were quantifiable (carnitine, acetylcarnitine, propionylcarnitine, hydroxybutyrylcarnitine, butyrylcarnitine, myristoleylcarnitine and palmitoylcarnitine), with carnitine and acetylcarnitine accounting for 83% and 13%, respectively, of all quantifiable carnitine species in average of all groups. No differences were observed in the plasma concentrations of the quantifiable carnitine species, except hydroxybutyrylcarnitine, one of the minor acylcarnitine species (≈ 1% of all species), which was 22% and 16% higher in group IM5 and group IM10, respectively, than in group CON (*P* < 0.05; Additional file 1: Table S8). Amongst the bile acids investigated in plasma, ten species were present in the plasma of pigs in quantifiable amounts. The major bile acids in plasma were hyodeoxycholic acid (HDCA), glycochenodeoxycholic acid (GCDCA) and chenodeoxycholic acid (CDCA) contributing to 63%, 17% and 16%, respectively, of all quantifiable plasma bile acids in average of all groups. Plasma concentrations of both, the major (HDCA, GCDCA and CDCA) and the minor bile acids [glycolithocholic acid, lithocholic acid, alpha-muricholic acid (MCA), omega-MCA, taurochenodeoxycholic acid, taurolithocholic acid and ursodeoxycholic acid] did not differ between the groups of pigs (Additional file 1: Table S9).

The sum of plasma concentrations of hexoses was not different across the three groups (group CON: 7560 ± 669 μmol/L, group IM5: 7302 ± 746 μmol/L, group IM10: 7100 ± 837 μmol/L, *P* = 0.405).

### Effect on hepatic and plasma TG and cholesterol concentrations and hepatic mRNA levels of lipogenic and cholesterogenic genes of the pigs

**Fig. 1:**
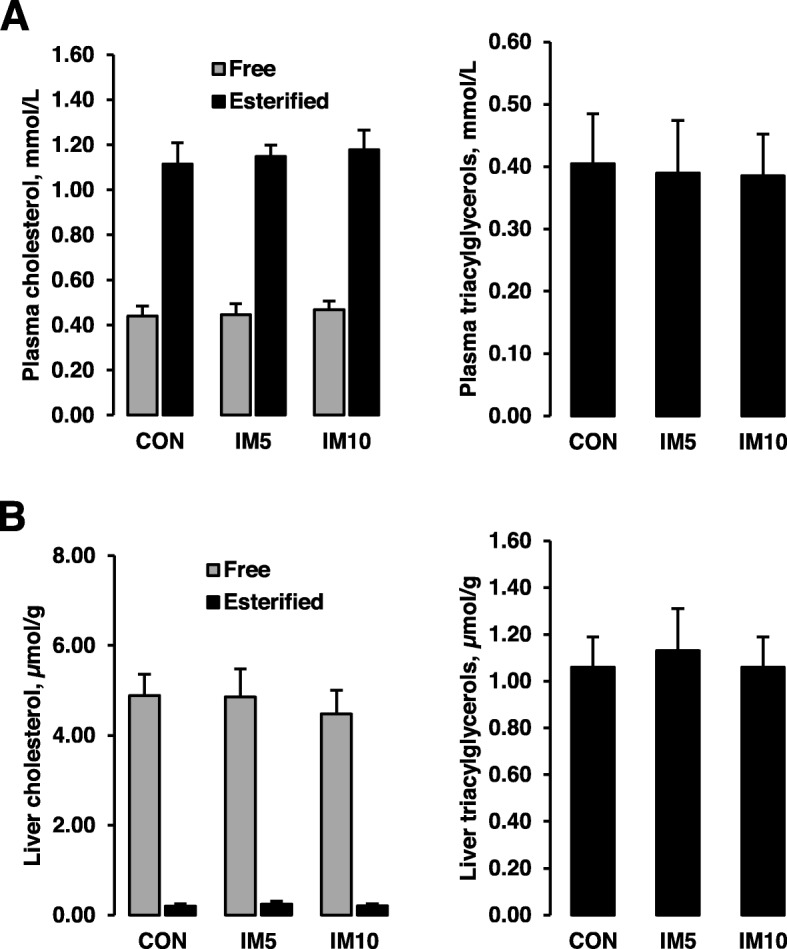
Concentrations of cholesterol and triacylglycerols in plasma (**a**) and liver (**b**) of pigs fed isonitrogenous diets without (CON) or with 5% insect meal (IM5) or 10% insect meal (IM10) for 4 weeks. Bars represent means ± SDs for *n* = 10 animals per group

Liver and plasma concentrations of TG and free and esterified cholesterol did not differ across the groups of pigs (Fig. 1). Hepatic mRNA levels of lipogenic genes (*ELOVL2*, *FADS1*, *FASN*, *SCD*) and cholesterogenic genes (*HMGCR*, *LDLR*, *MVK*, *SQLE*) were not different between the three groups of pigs (Additional file 1: Table S10). In addition, the hepatic mRNA level of the key gene involved in bile acid synthesis from cholesterol, *CYP7A1*, did not differ between groups (Additional file 1: Table S10).

### Effect on phospholipid and sphingolipid concentrations in liver and plasma of the pigs

In the liver, the concentrations of the main phospholipids (PC, PE, PI, PS), lysophospholipids (LPC, LPE) and sphingolipids (SM, Cer, HexCer) did not differ between the three groups (Table 7). Only the hepatic concentration of the minor phospholipids PG, PE P and PC O were higher in group IM5 and IM10 than in group CON (*P* < 0.05, Table 7). The critical PC:PE-ratio in the liver was higher in group IM10 than in group IM5 and group CON (*P* < 0.05, Table 7).

**Table 7:** Concentrations of phospholipid and sphingolipid classes in liver and plasma of pigs fed isonitrogenous diets without (CON) or with 5% insect meal (IM5) or 10% insect meal (IM10) for 4 weeks

	CON	IM5	IM10	*P*-value
Liver, μmol/g				
PC	16.2 ± 2.4	17.3 ± 2.0	17.1 ± 2.6	0.586
PE	8.36 ± 1.22	9.11 ± 1.18	8.32 ± 1.11	0.268
PI	5.05 ± 0.74	5.51 ± 0.87	5.12 ± 0.70	0.402
PS	2.47 ± 0.35	2.59 ± 0.34	2.53 ± 0.42	0.778
SM	1.10 ± 0.14	1.18 ± 0.18	1.13 ± 0.18	0.549
PE P	0.84 ± 0.21^b^	1.12 ± 0.18^a^	1.12 ± 0.21^a^	0.004
PC O	0.61 ± 0.13	0.65 ± 0.10	0.63 ± 0.15	0.759
LPC	0.12 ± 0.02	0.12 ± 0.02	0.13 ± 0.02	0.807
LPE	0.10 ± 0.02	0.11 ± 0.01	0.11 ± 0.02	0.342
PG	0.08 ± 0.02^b^	0.11 ± 0.02^a^	0.11 ± 0.03^a^	0.013
Cer	0.22 ± 0.05	0.22 ± 0.06	0.21 ± 0.05	0.915
HexCer	0.08 ± 0.02	0.08 ± 0.01	0.07 ± 0.02	0.127
PC:PE-ratio	1.94 ± 0.04^b^	1.91 ± 0.09^b^	2.05 ± 0.10^a^	0.006
Plasma, μmol/L				
PC	479 ± 51	522 ± 65	534 ± 38	0.083
SM	71.8 ± 8.8	77.5 ± 8.7	71.5 ± 7.0	0.236
PI	62.6 ± 7.1	66.0 ± 6.2	67.3 ± 6.7	0.314
LPC	46.0 ± 5.6	46.5 ± 4.2	46.7 ± 4.5	0.942
PC O	25.4 ± 4.2^b^	29.2 ± 4.8^b^	35.8 ± 3.8^a^	0.001
PE P	16.7 ± 3.3^c^	21.3 ± 3.7^b^	27.4 ± 3.4^a^	0.001
PE	9.72 ± 1.44	9.73 ± 1.68	10.05 ± 1.63	0.872
Cer	2.87 ± 0.43	2.64 ± 0.30	2.72 ± 0.34	0.403
HexCer	1.73 ± 0.30	1.39 ± 0.57	1.52 ± 0.38	0.223
PC:PE-ratio	48.7 ± 5.8	54.5 ± 7.3	52.2 ± 6.6	0.054

Values are means ± SDs for *n* = 10 pigs per group. Means without a common superscript letter differ, *P* < 0.05

Abbreviations: *Cer* ceramide, *CON* control group, *HexCer* hexosylceramide, *IM5* insect meal 5% group, *IM10* insect meal 10% group, *LPC* lysophosphatidylcholine, *LPE* lysophosphatidylethanolamine, *PC* phosphatidylcholine, *PC O* PC-ether, *PE* phosphatidylethanolamine, *PE P* PE-based plasmalogens, *PG* phosphatidylglycerol, *PI* phosphatidylinositol, *PS* phosphatidylserine, *SM* sphingomyelin

In plasma, the concentrations of the most abundant phospholipid PC and of other main phospholipids (PI, LPC, PE) and sphingolipids (SM, Cer, HexCer) were not different between the three groups. Only the plasma concentrations of PC O and PE P were higher in group IM10 than in group IM5 and group CON (*P* < 0.05, Table 7). The PC:PE-ratio in plasma did not differ across the groups (*P* < 0.05, Table 7).

### Effect on the composition of individual PC and PE species in the liver and composition of fatty acids of liver total lipids

**Fig. 2:**
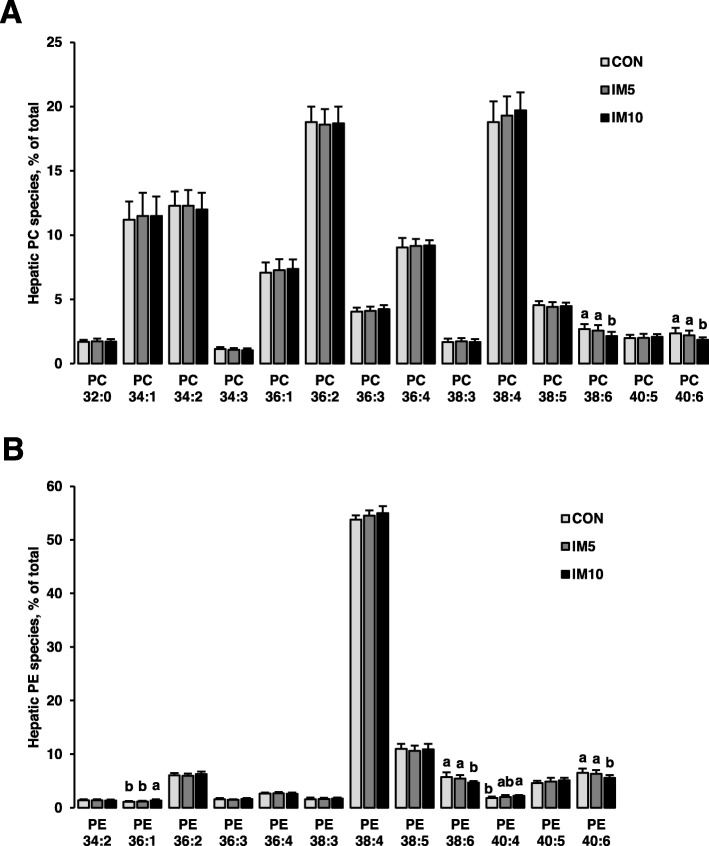
Composition of individual phosphatidylcholine (PC) species (**a**) and phosphatidylethanolamine (PE) species (**b**) in the liver of liver total lipids of pigs fed isonitrogenous diets without (CON) or with 5% insect meal (IM5) or 10% insect meal (IM10) for 4 weeks. Only PC and PE species > 1% are shown. Bars represent means ± SDs for *n* = 10 animals per group. Bars without a common letter differ, *P* < 0.05

PC and PE are the most abundant lipid species in the liver contributing to approximately 60% of hepatic total lipids. To evaluate the effect of IM on hepatic fatty acid metabolism, the composition of individual PC and PE species in the liver was determined. There were no differences in the percentages of all major PC and PE species in the liver between groups, with the exception of PC and PE species with 6 double bonds (PC 38:6, PC 40:6, PE 38:6, PE 40:6). The percentages of PC 38:6, PC 40:6, PE 38:6, and PE 40:6 were markedly lower in group IM10 than in group IM5 and group CON (*P* < 0.05, Fig. 2). No differences in this regard were found between group IM5 and group CON. In order to clarify if the reduction of PC and PE lipid species with 6 double bonds was due to a decrease of 22:6, fatty acid composition of liver total lipids was determined. As shown in Table 8, the percentage of 22:6n-3 was markedly reduced in group IM10 compared to the other groups (*P* < 0.05). The percentages of all other fatty acids in hepatic total lipids did not differ between groups. Hepatic ∆6 and ∆5 + 6 desaturation indices calculated from 20:3n-6/18:2n-6 ratio (∆6) and 20:4 n-6/18:2n-6 ratio (∆5 + 6) and 20:5n-3/18:3n-3 ratio (∆5 + 6) did not differ between groups. The ratio of 22:6n-3/22:5n-3 indicating the efficiency of endogenous production of 22:6n-3 from 22:5n-3 was lower in group IM10 compared to the other groups (*P* < 0.05).

**Table 8:** Fatty acid composition of liver total lipids of pigs fed isonitrogenous diets without (CON) or with 5% insect meal (IM5) or 10% insect meal (IM10) for 4 weeks

	CON	IM5	IM10	*P*-value
Fatty acids, g/100 g total FAME				
14:0	0.21 ± 0.12	0.27 ± 0.07	0.20 ± 0.15	0.774
15:0	0.41 ± 0.07	0.35 ± 0.06	0.39 ± 0.06	0.165
16:0	14.2 ± 1.1	14.2 ± 1.1	14.2 ± 0.9	0.628
16:1	0.42 ± 0.06	0.44 ± 0.04	0.44 ± 0.08	0.814
17:0	2.10 ± 1.20	1.62 ± 0.46	1.68 ± 0.29	0.637
17:1	0.47 ± 0.10	0.38 ± 0.09	0.38 ± 0.12	0.120
18:0	29.6 ± 0.8	29.6 ± 1.5	30.3 ± 1.0	0.323
18:1 *t*9	0.12 ± 0.05	0.15 ± 0.05	0.16 ± 0.06	0.096
18:1n-9	11.1 ± 1.0	11.2 ± 1.0	11.3 ± 1.6	0.914
18:2n-6	16.3 ± 1.1	16.2 ± 1.0	16.5 ± 1.6	0.796
18:3n-6	0.31 ± 0.09	0.24 ± 0.06	0.30 ± 0.05	0.104
18:3n-3	0.40 ± 0.10	0.33 ± 0.04	0.33 ± 0.10	0.163
20:0	0.27 ± 0.11	0.34 ± 0.24	0.32 ± 0.06	0.144
20:2n-6	0.49 ± 0.10	0.45 ± 0.04	0.48 ± 0.14	0.819
20:3n-6	0.73 ± 0.11	0.70 ± 0.07	0.63 ± 0.14	0.001
20:4n-6	18.2 ± 1.1	18.9 ± 0.8	18.2 ± 1.5	0.309
22:0	0.30 ± 0.06	0.30 ± 0.07	0.34 ± 0.08	0.276
20:5n-3	0.29 ± 0.09	0.24 ± 0.08	0.20 ± 0.06	0.067
22:5n-3	1.90 ± 0.34	1.92 ± 0.45	1.94 ± 0.41	0.980
22:6n-3	2.18 ± 0.42^a^	2.22 ± 0.27^a^	1.65 ± 0.36^b^	0.001
Product: substrate-ratios				
20:3n-6/18:2n-6	0.04 ± 0.01	0.04 ± 0.01	0.04 ± 0.01	0.075
20:4n-6/18:2n-6	1.12 ± 0.11	1.17 ± 0.06	1.11 ± 0.15	0.445
20:5n-3/18:3n-3	0.73 ± 0.18	0.72 ± 0.15	0.62 ± 0.14	0.224
22:6n-3/22:5n-3	1.20 ± 0.35^a^	1.23 ± 0.38^a^	0.88 ± 0.27^b^	0.049

Values are means ± SDs for *n* = 10 pigs per group. Means without a common superscript letter differ, *P* < 0.05

Abbreviations: *CON* control group; *FAME* fatty acid methyl esters; *IM5* insect meal 5% group; *IM10* insect meal 10% group

### Correlations between between significantly altered hepatic phospholipid parameters and significantly altered hepatic mRNA levels

In order to investigate the statistical relationships between hepatic mRNA levels of significantly altered genes and significantly altered hepatic parameters of lipid metabolism (PE P, PG and PC:PE-ratio), regression analysis was carried out. As shown in Additional file 1: Table S11, no significant correlations were found.

## Discussion

Apart from overcoming currently existing legal obstacles, one prerequisite for the inclusion of IM in feeding rations for monogastric farm animals is that animals´ performance is not impaired. In the present study, IM from *Tenebrio molitor* L. was included in the diets at two different levels (5% and 10%) as a source of protein in order to replace SEM on an isonitrogenous basis. The present study confirms results from several studies that IM can partially or completely replace conventional protein sources such as SEM in feeding rations for monogastric farm animals without strongly impairing their performance [4–12]. Even though BW gain was significantly reduced in group IM10 compared to the other groups, this effect should not be overstated regarding the limited number of pigs and the low number of observations, which is not sufficient for a classical performance trial. In addition, the unequal stocking density in each treatment group, which was found to affect BW gain and to interact with the diet effect, is not appropriate for a performance trial. Another important prerequisite for the use of IM as feed for monogastric farm animals is that it does not induce detrimental effects on intermediary metabolism. While some recent studies demonstrated that inclusion of IM from *Hermetia illucens* in feeding rations for broilers, ducks and pigs neither induces detrimental effects on blood chemical parameters nor causes any histopathological alterations [7, 8, 12–14], any in-depth investigations on the metabolic effects of IM in pigs were completely lacking. Against this background, the present study aimed to comprehensively describe for the first time the effects of IM on intermediary metabolism of pigs by means of omics-techniques.

One of the main findings obtained from genome-wide differential transcript profiling of liver and gastrocnemius muscle was that feeding IM causes only slight changes of the transcriptome of these tissues, which suggests a generally weak influence of IM on the metabolism of growing pigs. This conclusion is largely based on two observations: 1) In the liver, only four (*RDH16*, *CISH*, *HAMP*, *ACTG2*) and three genes (*TMEM52B*, *ADAD1*, *GTSF1*) were up- and downregulated, respectively, greater 2-fold, and, 2) in skeletal muscle, only one (*RDH16*) and two genes (*BTG2*, *ATF3*) were up- and downregulated, respectively, greater 2-fold in pigs fed the high inclusion level of IM (10%) compared to control pigs. In contrast to this very low number of strongly regulated genes, other dietary interventions, such as supplementation of *L*-carnitine, have been reported to profoundly affect the expression of a vast array of genes in metabolic tissues of growing pigs [43, 44]. Nevertheless, an interesting observation from transcriptomics of liver and skeletal muscle was the identification of *RDH16* as the most strongly up-regulated gene in both, liver (4.5-fold) and skeletal muscle (5.1-fold). In addition, *RDH16* was also found to be up-regulated, but less strong, in liver (3.1-fold) and skeletal muscle (4.2-fold) of pigs fed the low inclusion level of IM (5%) compared to control pigs. This indicates that induction of this gene by feeding IM occurs in a dose-dependent manner and is obviously not restricted to a particular metabolic tissue. Despite the function of the enzyme encoded by *RDH16* is well-known - retinol dehydrogenases catalyze the reversible oxidation/reduction of retinol and retinal–, the biological significance of this IM–specific effect on pigs’ metabolism remains obscure. Nevertheless, the observation from a recent study in pigs is at least noteworthy that *RDH16* and other genes involved in vitamin A metabolism are up-regulated in the liver of pigs with high feed efficiency, an important economic trait in growing pigs, compared to pigs with low feed efficiency [45].

To gain further insight into the biological meaning of IM-induced changes in the hepatic and skeletal muscle transcriptome, GSEA was performed with the differentially regulated genes, even though results have to be interpreted carefully considering the low filter settings applied for identifying differentially regulated genes (*P* < 0.05 and FC > 1.2-fold or FC < − 1.2-fold). While the overrepresented GO terms associated with the up-regulated genes in the liver, such as regulation of sequence-specific DNA binding transcription factor activity and transcription, DNA-templated, merely demonstrate that IM affects metabolism in an unspecific manner via altering gene expression, the overrepresented GO terms associated with the down-regulated genes in the liver (e.g., urea metabolic process, urea cycle, nitrogen cycle metabolic process, cellular response, arginine metabolic process) clearly indicate the modulation of a specific metabolic pathway, namely the urea cycle. The latter observation is presumably explained by the identification of genes encoding enzymes involved in the urea cycle, like argininosuccinate synthase 1, arginase 2 and arginase-1-like, amongst the down-regulated genes in pigs fed 10% IM. This may be indicative of a decreased urea synthesis in the liver of pigs fed IM as a consequence of a decreased or delayed transfer of dietary amino acids from the intestinal lumen into the portal vein. This assumption is indeed supported by the decreased ileal digestibility of amino acids in pigs fed 10% IM compared to control pigs. In line with these indications of a modulation of urea cycle by IM, targeted metabolomics of plasma of the pigs revealed that citrulline, an intermediate of the urea cycle, was increased in pigs of group IM10. The increased plasma citrulline concentration may be indicative of an accumulation of this intermediate as a result of the observed down-regulation of hepatic argininosuccinate synthase 1, which uses citrulline as substrate. One striking finding from transcript profiling of skeletal muscle was that G-protein-coupled receptor signaling pathway was the most enriched biological process term associated with the genes induced by IM. This is likely explained by the up-regulation of a significant number of transcripts encoding olfactory receptors (OLFRs), which comprise a large family of G protein-coupled receptors. Interestingly, despite OLFRs have been shown to facilitate perception of smell in the brain through sensing volatile chemicals (i.e. odorants) in the nasal olfactory epithelium, ectopic expression of OLFRs occurs also in non-chemosensory tissues like skeletal muscle [46], where these receptors are also functional and play a role in skeletal muscle development and regeneration [47, 48]. It is thus possible that IM modulates processes involved in skeletal muscle development and regeneration via regulating the expression of OLFRs. Future studies are warranted to clarify the biological consequences of the regulation of these genes in skeletal muscle of growing pigs by IM.

Apart from the mentioned increase of plasma citrulline, targeted metabolomics revealed further changes of some plasma amino acids (increases of alanine, glutamate, proline, serine, tyrosine and valine, decrease of asparagine) in pigs of group IM10. While some of these changes, in particular the lower changes, are probably the result of a different bioavailability of ingested amino acids due to slight variations in the amino acid concentrations between diets (e.g. alanine, proline), changes in other amino acids, like tyrosine and valine, whose plasma levels were increased by 50-60% in group IM10 compared to group CON, probably reflect a stronger impact of IM on the metabolism of these amino acids, of whatever kind and nature. With regard to plasma valine, its increase probably is not indicative of development of insulin resistance, because pigs were killed in the fed state. Future studies have to clarify the relevance of these alterations. In addition, plasma metabolomics revealed no effect of IM on the vast majority of quantifiable amino acid metabolites. The only exception was MetO, whose plasma concentration was elevated by 45% and 71% in pigs fed 5% and 10% IM, respectively. MetO is readily formed from methionine by ROS-dependent oxidation and reduced back to methionine by thioredoxin-dependent methionine sulfoxide reductases [49, 50]. In our recent study with obese Zucker rats [15], we have also made this striking finding of a dose-dependent increase of plasma MetO concentration by feeding IM using targeted plasma metabolomics (unpublished data). This indicates that this increase of plasma MetO concentration is probably an IM-specific effect, which obviously occurs in both rodents and pigs. Although the relevance of this finding is not clear, increased plasma levels of MetO might be indicative of the induction of oxidative stress by feeding IM, an effect which is generally considered critically due to the potential to cause damage to tissue components such as lipids, proteins and DNA. Nevertheless, whether or not IM indeed causes oxidative stress in tissues of pigs must be clarified using more established parameters of oxidative stress, such as malondialdehyde or nitrotyrosine. In this context, it is noteworthy that serum concentrations of malondialdehyde or nitrotyrosine were even reported to decrease with increasing inclusion level of IM from *Hermetia illucens* in feeding rations for ducks [14].

In addition to most of the amino acid metabolites, no effect of IM was observed on major and minor species of bile acids in plasma. Plasma bile acids represent a small fraction of bile acids, reabsorbed from the distal small intestine, which enter the systemic circulation due to bypassing their uptake out of liver sinusoids [51], whereas the main fraction of reabsorbed bile acids is taken up by the liver and secreted back to the biliary tract [52]. While bile acids have long been considered as simple detergents facilitating digestion of fat-soluble food components, bile acids have now been recognized as potent signaling molecules exhibiting regulatory effects on key metabolic tissues owing to activation of different nuclear hormone receptors [53–55]. Furthermore, almost no effects of IM were observed on plasma carnitine species by targeted metabolomics considering that the major carnitine species, free carnitine and acetylcarnitine, which made up > 96% of all carnitine species in plasma of the pigs, were not different across the groups. Only one of the minor carnitine species, hydroxybutyrylcarnitine, was slightly elevated (+ 20%) in plasma of pigs fed IM. Regarding this slight elevation and its low contribution to total carnitine species, we suggest that this finding is not of great biological significance. In connection with the weak changes of hepatic and skeletal muscle transcriptome the largely unaffected plasma metabolite levels between pigs fed IM and control pigs suggest that IM does not cause profound alterations of intermediary metabolism in healthy pigs. This assumption is also justified when considering the results obtained from lipidomics of liver and plasma of the pigs. In contrast to our recent studies in obese Zucker rats [15, 16], inclusion of IM into the diet had no effect on TG and cholesterol concentrations in plasma and liver of the pigs. However, the lack of a TG- and cholesterol-lowering effect as observed in obese Zucker rats, which exhibit severe hyperlipidemia and liver steatosis when compared to lean rats, is not surprising because physiologically normal levels of lipids in plasma and liver as found in young and metabolically healthy pigs are unlikely to be further reduced by dietary interventions. Thus, the unaltered plasma and liver TG and cholesterol concentrations rather indicate that IM has no detrimental effect on lipid metabolism in pigs. Also, contrary to the findings in obese rats, feeding IM exhibited no marked influence on phospholipid metabolism of the growing pigs regarding that the concentrations of the main phospholipids, such as PC, PE and PI, lysophospholipids and sphingolipids in liver and plasma did not differ between groups. The IM caused only slight increases of hepatic concentrations of PG and PE P – effects which should not be overestimated considering their small contribution of 0.3%, 2.7% and 2.2%, respectively, to total hepatic phospholipids and sphingolipids. Likewise, the only effects of IM on plasma phospholipid concentrations occurred on some of the minor phospholipid classes, PE P and PC O, which contributed only to 3.9, 2.8 and 1.7%, respectively, of total plasma phospholipids and sphingolipids in the pigs. Owing to the lack of effect of IM on the major phospholipid classes PC and PE, the hepatic and plasma PC:PE ratio was either not (plasma) or only slightly (liver) influenced by the IM. An increased hepatic PC:PE-ratio of greater 2 is considered critically [17], because it has been linked with the development of fatty liver disease [56, 57]. Despite the slight increase (+ 5%) of the hepatic PC:PE-ratio in pigs of group IM10 was statistically significant, we postulate that this increase is not of biological relevance considering the marked elevation (+ 35%) of the hepatic PC:PE-ratio in severely steatotic obese Zucker rats compared with non-steatotic lean Zucker rats [16]. In line with our assumption, there was no indication of fatty liver induction as evident from the observation that hepatic TG concentrations of the pigs of all groups were within the physiological range reported from others [58, 59]. With regard to individual phospholipid species, we observed that the percentages of PC and PE species with 6 double bonds were reduced in the liver of pigs of group IM10. This observation indicated that IM inhibits the desaturation of long-chain fatty acids. To reinforce this indication, we analyzed the fatty acid composition of hepatic total lipids, to which PC and PE contributed approximately 60%. This analysis revealed a decrease of the percentage of C22:6n-3 in the liver of pigs of group IM10, whereas the percentages of all other fatty acids did not differ between groups. Since we have recently observed that feeding IM inhibits hepatic desaturation of long-chain fatty acids in obese Zucker rats, we also calculated hepatic ∆6 and ∆5 + 6 desaturation indices from the product:substrate-ratios, which however did not differ across the groups. In contrast, the decreased ratio of 22:6n-3 to 22:5n-3 in the liver of pigs of group IM10 indicated that the formation of 22:6n-3 from 22:5n-3 is inhibited. 22:6 n-3 has long been assumed to be solely formed via a coupled microsomal-peroxisomal pathway. According to this pathway, 22:5n-3 is elongated into 24:5n-3 and subsequently ∆6-desaturated into 24:6n-3, which itself is retro-converted into 22:6n-3 via peroxisomal β-oxidation [60]. However, in 2015 it has been unequivocally demonstrated that 22:5n-3 can also be directly ∆4-desaturated into 22:6n-3 in human cells [61]. Since the existence of the coupled microsomal-peroxisomal pathway has not been excluded, the currently accepted view is that two putatively redundant biosynthetic pathways for the production of C22:6n-3 exist in mammalian cells. Thus, future research is necessary to clarify whether the reduced level of 22:6n-3 in response to feeding IM is due to an impairment of peroxisomal function or an inhibition of ∆4-desaturation. Apart from this, it is also possible that feeding of IM decreased the incorporation of 22:6n-3 into hepatic phospholipids.

Owing to the fact that protein-rich IM is a complex mixture of different chemical substances it is difficult to directly ascribe the metabolic effects of IM to its specific constituents. However, several metabolic effects induced by feeding specific dietary protein sources have been explained by specific amino acids and/or the presence of bioactive peptides [62]. Thus, differences in the concentration of certain amino acids and bioactive peptides between the CON diet and the IM diets may account for at least some of the effects observed. In addition, chitin –an intrinsic constituent of the insects' exoskeleton, which made up approximately 0.5% and 1% in the IM5 and IM10 diets - has been shown to exert nutrient-encapsulating and unspecific binding effects (e.g. sterols) in the intestine [63]. Such effects of chitin are causative for a reduction of nutrient digestibility and an increased fecal loss of sterols [64], both of which affects host metabolism. Since chitin is largely indigestible in the small intestine of monogastric animals, most of the chitin is expected to reach the large intestine, where it serves as a fermentation substrate for gut bacteria. It is well documented that short-chain fatty acids (SCFA) produced from carbohydrate and protein fermentation in the large intestine not only exhibit local effects on the intestinal mucosa, but also exert pronounced systemic effects through serving as substrates for main metabolic pathways (lipogenesis, gluconeogenesis). In addition, SCFA also act as signaling molecules regulating appetite, food intake and energy expenditure through activating various receptors for SCFA in key metabolic tissues and even the brain [65]. Finally, it has to be mentioned that inclusion of cellulose at levels of 2.8% and 5.7% in the IM5 and IM10 diets, respectively, was necessary to ensure isoenergetic replacement of SEM by IM. As a consequence, the concentration of crude fiber was also higher in the IM5 and IM10 diet than in the CON diet. Like chitin and other non-starch polysaccharides, cellulose has nutrient-encapsulating effects and increases, even though slightly, chyme viscosity, thereby, accelerating the transit of the intestinal chyme, which limits nutrient digestibility. Thus, the observed reduction of ileal digestibility of amino acids in pigs fed IM is probably not caused by chitin alone but also by cellulose. Moreover, cellulose also serves as a fermentation substrate for gut bacteria, thus, causing an increased production of SCFA, thereby, affecting host metabolism through providing additional energy substrates. Owing to this, it is important to note that the metabolic effects observed with feeding the IM diets cannot be solely attributed to specific constituents of IM, such as chitin, amino acids or bioactive peptides, but also to specific feed components of the IM5 and IM10 diets, such as cellulose. Accordingly, it is also not possible to ascribe specific effects induced by feeding the IM diets exclusively to the added cellulose.

## Conclusions

Comprehensive evaluation of the metabolic impact of dietary IM in growing pigs revealed a moderate differential regulation of a large number of transcripts in liver and skeletal muscle and alterations in the plasma concentrations of several amino acids (alanine, citrulline, glutamate, proline, serine, tyrosine, valine, asparagine) and the amino acid metabolite MetS. In contrast, no alterations in response to dietary IM were found in the plasma concentrations of major carnitine/acylcarnitine species and circulating bile acid species and in liver and plasma concentrations of main lipid classes (TG, cholesterol, phospholipids, lysophospholipids, sphingolipids). The percentages of all individual PC and PE species in the liver showed no differences between groups, except those with 6 double bonds, which were lower in group IM10 than in CON. Based on these observations, we conclude that partial or complete replacement of SEM by IM in the feeding ration has overall less impact on the intermediary metabolism of growing pigs. Thus, IM from *Tenebrio molitor* L. can be regarded as a safe source of protein in piglets which does not cause adverse effects on metabolism.

## Supplementary information

**Additional file 1 Table S1.** Chemical composition of insect meal from *Tenebrio molitor* L. **Table S2.** Characteristics of gene-specific primers used for qPCR analysis in liver and skeletal muscle. **Table S3.** Prececal digestibilities of amino acids in pigs fed isonitrogenous diets without (CON) or with 10% insect meal (IM10) for 4 weeks. **Table S4.** Up- and down-regulated genes in the liver of pigs of group IM10 compared with group CON.* **Table S5.** qPCR validation of microarray data for selected differentially expressed transcripts (FC > 1.2 or < − 1.2, *P* < 0.05) in the liver of pigs of group IM10 compared with group CON.* **Table S6.** Up- and down-regulated genes in the gastrocnemius muscle of pigs of group IM10 compared with group CON.* **Table S7.** qPCR validation of microarray data for selected differentially expressed transcripts (FC > 1.2 or < − 1.2, *P* < 0.05) in gastrocnemius muscle of pigs of group IM10 compared with group CON.* **Table S8.** Plasma concentrations of carnitine species of pigs fed isonitrogenous diets without (CON) or with 5% insect meal (IM5) or 10% insect meal (IM10) for 4 weeks. **Table S9.** Plasma concentrations of bile acids of pigs fed isonitrogenous diets without (CON) or with 5% insect meal (IM5) or 10% insect meal (IM10) for 4 weeks. **Table S10.** Hepatic mRNA levels of genes involved in fatty acid, cholesterol and bile acid synthesis in the liver of pigs fed isonitrogenous diets without (CON) or with 5% insect meal (IM5) or 10% insect meal (IM10) for 4 weeks. **Table S11.** Correlation analysis between significantly altered hepatic phospholipid parameters and significantly altered hepatic mRNA levels in pigs fed isonitrogenous diets without (CON) or with 5% insect meal (IM5) or 10% insect meal (IM10) for 4 weeks.

## Data Availability

All data generated or analyzed during this study are included in this published article and its supplementary information files.
