# Supplementary material for: Comprehensive evaluation of the metabolic effects of insect meal from Tenebrio molitor L. in growing pigs by transcriptomics, metabolomics and lipidomics
Source: J Anim Sci Biotechnol. 2020 Mar 4;11:20. doi: 10.1186/s40104-020-0425-7 (PMC7055059; doi:10.1186/s40104-020-0425-7)
Supplement: Supplementary file 1 — Additional file 1 Table S1. Chemical composition of insect meal from Tenebrio molitor L. Table S2. Characteristics of gene-specific primers used for qPCR analysis in liver and skeletal muscle. Table S3. Prececal digestibilities of amino acids in pigs fed isonitrogenous diets without (CON) or with 10% insect meal (IM10) for 4 weeks. Table S4. Up- and down-regulated genes in the liver of pigs of group IM10 compared with group CON.* Table S5. qPCR validation of microarray data for selected differentially expressed transcripts (FC > 1.2 or < − 1.2, P < 0.05) in the liver of pigs of group IM10 compared with group CON.* Table S6. Up- and down-regulated genes in the gastrocnemius muscle of pigs of group IM10 compared with group CON.* Table S7. qPCR validation of microarray data for selected differentially expressed transcripts (FC > 1.2 or < − 1.2, P < 0.05) in gastrocnemius muscle of pigs of group IM10 compared with group CON.* Table S8. Plasma concentrations of carnitine species of pigs fed isonitrogenous diets without (CON) or with 5% insect meal (IM5) or 10% insect meal (IM10) for 4 weeks. Table S9. Plasma concentrations of bile acids of pigs fed isonitrogenous diets without (CON) or with 5% insect meal (IM5) or 10% insect meal (IM10) for 4 weeks. Table S10. Hepatic mRNA levels of genes involved in fatty acid, cholesterol and bile acid synthesis in the liver of pigs fed isonitrogenous diets without (CON) or with 5% insect meal (IM5) or 10% insect meal (IM10) for 4 weeks. Table S11. Correlation analysis between significantly altered hepatic phospholipid parameters and significantly altered hepatic mRNA levels in pigs fed isonitrogenous diets without (CON) or with 5% insect meal (IM5) or 10% insect meal (IM10) for 4 weeks. [file 40104_2020_425_MOESM1_ESM.docx]

**Table S1**

Chemical composition of insect meal from *Tenebrio molitor* L.

|  | Insect meal |
| --- | --- |
| *Crude nutrients* |  |
| Crude protein, % of DM | 74.0 |
| Crude fat, % of DM | 10.3 |
| Crude fiber, % of DM | 9.5 |
| Crude ash, % of DM | 5.5 |
| Chitin, % of DM | 9.7 |
| Gross energy, MJ/kg DM | 23.3 |
| *Amino acids (g/kg FM)* |  |
| Alanine | 47.3 |
| Arginine | 32.6 |
| Aspartic acid | 58.0 |
| Cysteine | 5.4 |
| Glutamic acid | 82.3 |
| Glycine | 33.2 |
| Histidine | 17.1 |
| Isoleucine | 27.1 |
| Leucine | 48.5 |
| Lysine | 33.1 |
| Methionine | 7.7 |
| Phenylalanine | 22.9 |
| Proline | 54.5 |
| Serine | 29.6 |
| Threonine | 26.6 |
| Tryptophan | 7.5 |
| Tyrosine | 45.2 |
| Valine | 37.7 |
| *Fatty acids^1^ (g/100 g total FAME)* |  |
| 12:0 | 0.3 |
| 14:0 | 2.4 |
| 16:0 | 15.5 |
| 16:1n-9 | 0.7 |
| 18:0 | 4.5 |
| 18:1n-9 | 35.0 |
| 18:2n-6 | 39.2 |
| 18:3n-3 | 1.5 |
| 20:0 | 0.1 |

^1^Only fatty acids with concentrations ≥ 0.1 g/100 g total fatty acids are shown.

Abbreviations: DM, dry matter; FAME, fatty acid methyl esters; FM, fresh matter.

**Table S2**

Characteristics of gene-specific primers used for qPCR analysis in liver and skeletal muscle.

| Gene | Forward (5` to 3`)  Reverse (5` to 3`) | Annealing temperature (°C) | PCR product size (bp) | NCBI GeneBank accession no. | Slope | *R^2^* | E |
| --- | --- | --- | --- | --- | --- | --- | --- |
| ***Liver*** |  |  |  |  |  |  |  |
| *Reference genes* | |  |  |  |  |  |  |
| *GAPDH* | GTCGGAGTGAACGGATTTGG  TGGAAGATGGTGATGGCCTT | 60 | 218 | NM_001206359 | -3.34 | 0.995 | 1.99 |
| *GPI* | CACGAGCACCGCTCTGACCT  CCACTCCGGACACGCTTGCA | 64 | 365 | NM_214330 | -3.55 | 0.984 | 1.91 |
| *SDHA* | CTACGCCCCCGTCGCAAAGG AGTTTGCCCCCAGGCGGTTG | 64 | 380 | XM_021076930 | -3.63 | 0.989 | 1.89 |
| *Target genes* | |  |  |  |  |  |  |
| *ACTG2* | GCCATTCAAGCTGTGCTCTCC  TCGCACGATTTCTCGTTCAGC | 60 | 228 | XM_021087371 | -3.55 | 0.994 | 1.91 |
| *ADAD1* | TGTGTCAATGGCAAGTCGGC  GGAGCCATAGCCATGTTGCTG | 62 | 187 | XM_003357037 | -3.18 | 0.999 | 2.06 |
| *APCS* | TTTGGGTCTCTGTCCTCGCC  TTCACATGGTCAGTGGCAGACT | 60 | 103 | NM_213887 | -3.53 | 0.998 | 1.92 |
| *CCL14* | GATTGCTGCTTCACCCACCTG  GGGTTGGCACAAACGAAACG | 58 | 134 | NM_001256775 | -3.51 | 0.996 | 1.93 |
| *CISH* | CCTCGTCATCAACCGTCTGGT  AGCTGGAAAGGGTACTGTCGG | 60 | 90 | NM_001315788 | -3.58 | 0.999 | 1.90 |
| *CLEC4F* | CAGCGGTTCTGAGGGCATCT  TGTCTGGCTGATTCTCGCTCC | 60 | 92 | XM_021087395 | -3.59 | 0.992 | 1.90 |
| *CUEDC1* | CGCCCTGGAGAGAGATCGAC  AGGCATCTTCGGACACAGCA | 62 | 137 | NM_001243919 | -3.50 | 0.951 | 1.93 |
| *CYP7A1* | AATTTGCTTGCCGCAGCCAT  TGCTTTCTGTGCATCGTGCC | 60 | 186 | NM_001005352 | -3.36 | 0.995 | 1.99 |
| *CYP26A1* | AACCTCATCCACGCTCGCAT  CCCTCTCCCATGAGTGCTCG | 60 | 124 | NM_001315792 | -3.61 | 0.959 | 1.89 |
| *DUSP6* | GTAAGTTCCAAGCCGAGTTCGC  AGAAGGCTGGCTGTTGGACA | 61 | 206 | NM_001267842 | -3.57 | 0.996 | 1.90 |
| *ELOVL2* | TCCCGTGCGGACAGAGTTTC  TGGGTGATGGTGAGCACGAA | 60 | 166 | XM_021100179 | -3.33 | 0.998 | 1.99 |
| *FADS1* | ACGTCACTGCCTGGCTCATT  CAGTTGGGCTTGGCATGGTG | 60 | 247 | NM_001113041 | -3.30 | 0.995 | 2.01 |
| *FASN* | AGCCTAACTCCTCGCTGCAAT  TCCTTGGAACCGTCTGTGTTC | 60 | 196 | NM_001099930 | -3.14 | 0.991 | 2.08 |
| *FCER1A* | TGGCAGCAGTCATCCAGGAAT  CATCTGTATTTGCCGCTGTCCC | 60 | 211 | XM_021089597 | -3.24 | 0.983 | 2.04 |
| *GPRIN3* | TCGACGTTGCCTCCAGATCC  GGAACGTCCTCGGGCTCATT | 59 | 116 | XM_021101761 | -3.50 | 0.999 | 1.93 |
| *GTSF1* | GACCCTGGCTGAGAGCACAT  TGCTTGCAGGGCTGTTGTTG | 60 | 131 | XM_003126205 | -3.50 | 0.970 | 1.93 |
| *HAMP* | GCTCCGTTCTCCCATCCCAG  AGATTGCTTTGCGACAGCAGC | 62 | 159 | NM_214117 | -3.59 | 0.995 | 1.90 |
| *HMGCR* | GGTCAGGATGCGGCACAGAACG  GCCCCACGGTCCCGATCTCTATG | 64 | 127 | NM_001122988 | -3.47 | 0.997 | 1.94 |
| *ID1* | GCAGGTGAACGTGCTCCTCTA  CTGCTCACCTTGCGGTTCTG | 60 | 93 | NM_001244700 | -3.43 | 0.998 | 1.96 |
| *LDLR* | TGCGAAGATATCGACGAGTG  TACGGTCCAGGGTCATCTTC | 58 | 196 | NM_001206354 | -3.39 | 0.999 | 1.97 |
| *MVK* | TGCGAGGAGATCCCAAACCC  AGTCGGCAGCCTCTTCAAGG | 60 | 207 | XM_001929184 | -3.25 | 0.995 | 2.03 |
| *NOV* | GGAGTGCGTGTTCCAAGAGC  TGACTTGGTGGTGCGGAGAC | 60 | 173 | XM_001927050 | -3.42 | 0.996 | 1.96 |
| *SCD* | TCGTCGCCACCTTTCTTCGT  CCTCACCCACAGCTCCCAAT | 60 | 147 | NM_213781 | -3.56 | 0.999 | 1.91 |
| *SLC6A8* | TGGTCTGGCTTTCATCGCCT  CGATGACAAAGCAGAGGGCG | 60 | 227 | NM_001177327 | -3.50 | 0.990 | 1.93 |
| *SLPI* | TCAAATGCCTGAACCCTGTTGC  TGGCTGTCTGTCTTGCAGTGA | 59 | 121 | NM_213870 | -3.21 | 0.955 | 2.05 |
| *SMAD7* | GTGCAAAGTGTTCAGGTGGC  CACAGCATCTGGACAGTCAGT | 58 | 208 | NM_001244175 | -3.46 | 0.962 | 1.94 |
| *SQLE* | TGTGAATGTCCTTGCTCAGGCT  ATCAGCGCAACTGGACCACTAA | 60 | 132 | NM_001101026 | -3.35 | 0.997 | 1.99 |
| *TAGLN* | GATGGACACTACCGTGGCGA  GAGGTCGTCCGTAGCCTGTC | 60 | 166 | NM_001244150 | -3.60 | 0.993 | 1.90 |
| *TFRC* | ACGGACAGCAACAGCTTGGTA  GCATTTGCCACCTTCTCAGCAA | 60 | 203 | NM_214001 | -3.28 | 0.987 | 2.02 |
|  |  |  |  |  |  |  |  |
| ***Skeletal muscle*** | |  |  |  |  |  |  |
| *Reference genes* | |  |  |  |  |  |  |
| *ATP5MC1* | CAGTCACCTTGAGCCGGGCGA  TAGCGCCCCGGTGGTTTGC | 64 | 94 | NM_001025218 | -3.28 | 0.993 | 2.02 |
| *GAPDH* | GTCGGAGTGAACGGATTTGG  TGGAAGATGGTGATGGCCTT | 60 | 218 | NM_001206359 | -3.32 | 0.994 | 2.00 |
| *RPS9* | GTCGCAAGACTTATGTGACC  AGCTTAAAGACCTGGGTCTG | 60 | 325 | XM_021094878 | -3.55 | 0.997 | 1.91 |
| *SDHA* | CTACGCCCCCGTCGCAAAGG  AGTTTGCCCCCAGGCGGTTG | 64 | 380 | XM_021076930 | -3.56 | 0.998 | 1.91 |
|  |  |  |  |  |  |  |  |
| *Target genes* | |  |  |  |  |  |  |
| *ABRA* | CCATCGCCAGAATCAAACGCC  CACTTGGCTGTATTTCCGCTGG | 60 | 92 | NM_001135960 | -3.46 | 1.000 | 1.94 |
| *ARG2* | GCTGTAAGGGAAGCTGGCTTGA  GCCACCTGACACCGCTCTAC | 60 | 189 | XM_001928679 | -3.28 | 0.995 | 2.02 |
| *ATF3* | AGTCAGTGCCTCTGCCATCG  GGCTACCTCGGCTTTCGTGA | 60 | 208 | XM_003482749 | -3.21 | 0.996 | 2.05 |
| *ATF5* | AGAGACCAGAACAAGTCCGCA  TGACATACTGGATCTCCCGCTC | 61 | 151 | XM_013998679 | -3.47 | 1.000 | 1.94 |
| *BEST3* | CCCTGCTCATCTTCCGCTCA  ACCAGGAGCGAAATCGGTTCA | 60 | 255 | XM_003481658 | -3.27 | 0.999 | 2.02 |
| *BTG2* | CTGCCGCTGTAGGTTTCCTCT  TGGTTGATGCGAATGCAGCG | 61 | 175 | NM_001097505 | -3.58 | 1.000 | 1.90 |
| *CLK1* | GAGGAGGGTCACCTGATCTGTC  GCGGAATGTACTGCTAGGGTCT | 60 | 237 | NM_001123099 | -3.58 | 0.998 | 1.90 |
| *ENHO* | CGCTCTGCCAACATCGACTC  CTTCATGGCTGGGCTTCTGG | 60 | 100 | NM_001164858 | -3.51 | 0.995 | 1.93 |
| *GBE1* | CCCGTACCTGAAGCCCTACG  CATCAGCACATCTGTGGATGCC | 60 | 146 | XM_021070783 | -3.36 | 1.000 | 1.98 |
| *GPA33* | TGAACCAGGAGCGTCATCAGG  ACGTTCATGGAGGGTGGTCTG | 61 | 163 | XM_005663142 | -3.37 | 0.994 | 1.98 |
| *IFRD1* | TCGCTGCGGGTGAATCTCTG  ACCGCCCTCAGGATGTCTCT | 60 | 187 | NM_001007519 | -3.42 | 0.997 | 1.96 |
| *JUN* | CCAGTCCAGTAACGGGCACA  CCCTCCTGCTCGTCAGTCAC | 60 | 84 | NM_213880 | -3.45 | 1.000 | 1.95 |
| *LOC100512656* | CGGCTACTGCCTGTCCAAGT  AACACCTCAGCGGCAGTCAT | 60 | 135 | XM_021091815 | -3.58 | 0.994 | 1.90 |
| *OCIAD2* | GCCACCACTTAGCAAGCAGAG  CAAACCTCGGGTTAGCTGCC | 60 | 197 | XM_005666640 | -3.52 | 0.998 | 1.92 |
| *OLFM3* | GCACAGCGAAGCCTGGAGTA  ACACAGCCCACAACCCGATT | 60 | 109 | XM_021090127 | -3.20 | 0.992 | 2.05 |
| *PDZD9* | TTACCCTGCGAGGAGACCAC  TCGGAGGTAGTGGAGACGGA | 60 | 207 | XM_021086490 | -3.29 | 0.996 | 2.01 |
| *PFKFB3* | GAAGCTCTCCAGCCCGGATT  GGCTGGTAGCTGGCTTCGTA | 61 | 96 | XM_013980318 | -3.15 | 0.997 | 2.08 |
| *RBP7* | TCCACACCAACAGCAGCCTAA  GTGGGTCCAGCCTCTGTTCTT | 60 | 173 | NM_001145222 | -3.22 | 0.996 | 2.04 |
| *SOX2* | AGAACAGCCCAGACCGAGTT  CCGTCTGCTGATCTCCGAGT | 61 | 119 | NM_001123197 | -3.33 | 0.991 | 2.00 |
| *VAV1* | ATCAACCTGCGGGAGGTCAAC  AAGAGTTCACTCCGCTTGAGGC | 60 | 119 | NM_001267833 | -3.42 | 0.990 | 1.96 |

**Table S3**

Prececal digestibilities of amino acids in pigs fed isonitrogenous diets without (CON) or with 10% insect meal (IM10) for 4 weeks.

|  | CON | IM10 |
| --- | --- | --- |
| *Essential amino acids, %* | |  |
| Arginine | 80.9 ± 6.0^a^ | 70.4 ± 8.1^b^ |
| Histidine | 77.9 ± 5.9^a^ | 64.1 ± 8.8^b^ |
| Isoleucine | 75.2 ± 6.8^a^ | 62.8 ± 9.1^b^ |
| Leucine | 76.2 ± 6.7^a^ | 64.7 ± 8.9^b^ |
| Lysine | 81.7 ± 5.1^a^ | 74.4 ± 6.5^b^ |
| Methionine | 83.3 ± 7.3 | 76.6 ± 7.0 |
| Phenylalanine | 77.1 ± 6.8^a^ | 63.2 ± 9.8^b^ |
| Threonine | 74.0 ± 6.6^a^ | 62.8 ± 8.8^b^ |
| Tryptophan | 73.9 ± 6.5^a^ | 62.1 ± 9.3^b^ |
| Valine | 73.5 ± 7.0^a^ | 63.0 ± 9.0^b^ |
| *Non-essential amino acids, %* | |  |
| Alanine | 66.3 ± 9.0^a^ | 56.3 ± 10.9^b^ |
| Aspartic acid | 69.4 ± 7.7^a^ | 43.1 ± 11.0^b^ |
| Cysteine | 61.4 ± 14.4^a^ | 48.4 ± 11.6^b^ |
| Glutamic acid | 85.3 ± 4.3^a^ | 77.8 ± 5.9^b^ |
| Glycine | 53.7 ± 15.7 | 45.3 ± 10.9 |
| Proline | 78.1 ± 5.6^a^ | 70.8 ± 6.9^b^ |
| Serine | 73.8 ± 6.6^a^ | 59.0 ± 9.5^b^ |
| Tyrosine | 72.3 ± 7.1^a^ | 61.3 ± 9.4^b^ |

Values are means ± SDs for *n* = 10 pigs per group. Means without a common superscript letter differ, *P* < 0.05.

Abbreviations: CON, control group; IM10, insect meal 10% group.

**Table S4**

Up- and down-regulated genes in the liver of pigs of group IM10 compared with group CON.*

| Gene symbol | Gene description | | IM5 vs. CON | | IM10 vs. CON | |
| --- | --- | --- | --- | --- | --- | --- |
|  |  | | FC | *P*-value | FC | *P*-value |
| *Up-regulated* |  | |  |  |  |  |
| *RDH16/LOC100512656* | *retinol dehydrogenase 16* | | 3.12 | 0.169 | 4.45 | 0.033 |
| *CISH* | *cytokine inducible SH2-containing protein* | | 2.11 | 0.040 | 3.45 | 0.004 |
| *HAMP* | *hepcidin antimicrobial peptide* | | 1.94 | 0.088 | 2.97 | 0.007 |
| *ACTG2* | *actin, gamma 2, smooth muscle, enteric* | | 1.97 | 0.223 | 2.87 | 0.037 |
| *ID1* | *inhibitor of DNA binding 1, dominant negative helix-loop-helix protein* | | 1.23 | 0.248 | 1.60 | 0.007 |
| *LOC102166306* | *alpha-fetoprotein-like* | | 1.31 | 0.068 | 1.53 | 0.042 |
| *FCER1A* | *Fc fragment of IgE, high affinity I, receptor for; alpha polypeptide* | | 1.42 | 0.008 | 1.52 | 0.000 |
| *MASTL* | *microtubule associated serine/threonine kinase-like* | | 1.29 | 0.149 | 1.46 | 0.019 |
| *TFRC* | *transferrin receptor* | | 1.12 | 0.532 | 1.45 | 0.020 |
| *CUEDC1* | *CUE domain containing 1* | | 1.37 | 0.066 | 1.42 | 0.035 |
| *LPIN1* | *lipin 1* | | 1.27 | 0.035 | 1.41 | 0.006 |
| *NOV* | *nephroblastoma overexpressed* | | 1.38 | 0.003 | 1.41 | 0.011 |
| *INSIG1* | *insulin induced gene 1* | | 1.18 | 0.281 | 1.38 | 0.033 |
| *TRIM15* | *tripartite motif containing 15* | | 1.25 | 0.043 | 1.37 | 0.004 |
| *SMAD7* | *SMAD family member 7* | | 1.07 | 0.426 | 1.37 | 0.007 |
| *BHLHE40* | *basic helix-loop-helix family, member e40* | | 1.20 | 0.029 | 1.36 | 0.000 |
| *LOC100154932* | *putative olfactory receptor 2B8* | | -1.01 | 0.853 | 1.36 | 0.039 |
| *TAGLN* | *transgelin* | | 1.51 | 0.100 | 1.34 | 0.025 |
| *ZNRD1* | *zinc ribbon domain containing 1* | | 1.23 | 0.271 | 1.33 | 0.043 |
| *LOC100517360* | *PR domain zinc finger protein 10* | | 1.11 | 0.287 | 1.33 | 0.007 |
| *LOC100624993* | *olfactory receptor 52D1-like* | | -1.00 | 0.993 | 1.33 | 0.006 |
| *NQO1* | *NAD(P)H dehydrogenase, quinone 1* | | 1.24 | 0.041 | 1.32 | 0.018 |
| *ITM2A* | *integral membrane protein 2A* | | 1.28 | 0.002 | 1.32 | 0.001 |
| *FOXA2* | *forkhead box A2* | | 1.20 | 0.014 | 1.31 | 0.008 |
| *LOC100151915* | *olfactory receptor 6C75-like* | | 1.15 | 0.195 | 1.31 | 0.025 |
| *DND1* | *DND microRNA-mediated repression inhibitor 1* | | 1.15 | 0.051 | 1.31 | 0.013 |
| *CCDC80* | *coiled-coil domain containing 80* | | 1.06 | 0.666 | 1.31 | 0.001 |
| *MAP3K13* | *mitogen-activated protein kinase kinase kinase 13* | | 1.11 | 0.240 | 1.31 | 0.019 |
| *CHKA* | *choline kinase alpha* | | 1.20 | 0.076 | 1.31 | 0.025 |
| *BPIFC* | *BPI fold containing family C* | | 1.10 | 0.532 | 1.30 | 0.027 |
| *PIWIL3* | *piwi-like RNA-mediated gene silencing 3* | | 1.06 | 0.426 | 1.30 | 0.014 |
| *CYP1A2* | *cytochrome P450, family 1, subfamily A, polypeptide 2* | | 1.32 | 0.008 | 1.30 | 0.015 |
| *FZD4* | *frizzled class receptor 4* | | 1.20 | 0.010 | 1.29 | 0.004 |
| *CYP7A1* | *cytochrome P450, family 7, subfamily A, polypeptide 1* | | 1.00 | 0.976 | 1.29 | 0.039 |
| *LIPG* | *lipase, endothelial* | | 1.16 | 0.174 | 1.29 | 0.012 |
| *LOC102161647* | *trichohyalin* | | -1.01 | 0.942 | 1.28 | 0.005 |
| *LOC100523525* | *claudin-34-like* | | 1.09 | 0.348 | 1.28 | 0.005 |
| *TBKBP1* | *TBK1 binding protein 1* | | 1.15 | 0.088 | 1.28 | 0.011 |
| *TTC7B* | *tetratricopeptide repeat domain 7B* | | 1.19 | 0.019 | 1.28 | 0.002 |
| *ENTPD5* | *ectonucleoside triphosphate diphosphohydrolase 5* | | 1.26 | 0.072 | 1.27 | 0.038 |
| *KIT* | *v-kit Hardy-Zuckerman 4 feline sarcoma viral oncogene homolog* | | 1.40 | 0.035 | 1.27 | 0.046 |
| *CYP2A19* | *cytochrome P450 2A19* | | 1.19 | 0.033 | 1.27 | 0.001 |
| *KIAA1551* | *KIAA1551 ortholog* | | 1.11 | 0.144 | 1.27 | 0.034 |
| *SKIDA1* | *SKI/DACH domain containing 1* | | 1.07 | 0.463 | 1.27 | 0.016 |
| *ID4* | *inhibitor of DNA binding 4, dominant negative helix-loop-helix protein* | | 1.12 | 0.160 | 1.27 | 0.009 |
| *PDZRN3* | *PDZ domain containing ring finger 3* | | 1.12 | 0.223 | 1.27 | 0.041 |
| *ZCCHC16* | *zinc finger, CCHC domain containing 16* | | 1.05 | 0.522 | 1.26 | 0.002 |
| *LOC100157987* | *zinc finger and SCAN domain-containing protein 21-like* | | 1.13 | 0.004 | 1.26 | 0.003 |
| *LOC100626014* | *calcium/calmodulin-dependent protein kinase type II subunit alpha* | | 1.19 | 0.013 | 1.26 | 0.000 |
| *AK5* | *adenylate kinase 5* | | 1.12 | 0.213 | 1.25 | 0.022 |
| *PRPH* | *peripherin* | | 1.39 | 0.037 | 1.25 | 0.047 |
| *GRB7* | *growth factor receptor-bound protein 7* | | 1.11 | 0.402 | 1.25 | 0.023 |
| *KLF10* | *Kruppel-like factor 10* | | 1.14 | 0.260 | 1.25 | 0.008 |
| *KLF10* | *Kruppel-like factor 10* | | 1.14 | 0.260 | 1.25 | 0.008 |
| *LOC100625368* | *lengsin-like* | | 1.10 | 0.194 | 1.25 | 0.025 |
| *ATG14* | *autophagy related 14* | | 1.12 | 0.054 | 1.25 | 0.004 |
| *FAM214A* | *family with sequence similarity 214, member A* | | 1.18 | 0.018 | 1.25 | 0.041 |
| *SURF6* | *surfeit 6* | | 1.15 | 0.049 | 1.25 | 0.001 |
| *FBXO32* | *F-box protein 32* | | 1.30 | 0.005 | 1.25 | 0.019 |
| *EBF4* | *early B-cell factor 4* | | 1.17 | 0.029 | 1.25 | 0.007 |
| *LOC100524490* | *olfactory receptor 2AE1-like* | | 1.20 | 0.031 | 1.24 | 0.016 |
| *ATIC* | *5-aminoimidazole-4-carboxamide ribonucleotide formyltransferase/IMP cyclohydrolase* | | 1.29 | 0.034 | 1.24 | 0.033 |
| *CSTL1* | *cystatin-like 1* | | 1.22 | 0.018 | 1.24 | 0.001 |
| *PIR* | *pirin (iron-binding nuclear protein)* | | 1.15 | 0.007 | 1.24 | 0.027 |
| *KIF20B* | *kinesin family member 20B* | | 1.15 | 0.065 | 1.24 | 0.005 |
| *MOB3C* | *MOB kinase activator 3C* | | 1.45 | 0.004 | 1.24 | 0.012 |
| *DLG5* | *discs, large homolog 5 (Drosophila)* | | 1.13 | 0.192 | 1.24 | 0.019 |
| *TDP2* | *tyrosyl-DNA phosphodiesterase 2* | | 1.13 | 0.221 | 1.23 | 0.032 |
| *LOC100627357* | *olfactory receptor 13A1-like* | | 1.16 | 0.101 | 1.23 | 0.010 |
| *NPTXR* | *neuronal pentraxin receptor* | | 1.04 | 0.490 | 1.23 | 0.036 |
| *SLC5A2* | *solute carrier family 5 (sodium/glucose cotransporter), member 2* | | 1.04 | 0.406 | 1.23 | 0.003 |
| *XKR9* | *XK, Kell blood group complex subunit-related family, member 9* | | 1.03 | 0.685 | 1.23 | 0.001 |
| *AQP2* | *aquaporin 2 (collecting duct)* | | 1.19 | 0.079 | 1.23 | 0.040 |
| *LOC100523107* | *melanoma-associated antigen D4* | | 1.11 | 0.200 | 1.23 | 0.046 |
| *IFN-ALPHA-4* | *interferon-alpha-4* | | 1.12 | 0.205 | 1.23 | 0.019 |
| *PI15* | *peptidase inhibitor 15* | | 1.11 | 0.187 | 1.23 | 0.035 |
| *CCDC91* | *coiled-coil domain containing 91* | | 1.11 | 0.295 | 1.23 | 0.042 |
| *SLC5A12* | *solute carrier family 5 (sodium/monocarboxylate cotransporter), member 12* | | 1.07 | 0.275 | 1.23 | 0.010 |
| *OLFML2A* | *olfactomedin-like 2A* | | 1.13 | 0.016 | 1.23 | 0.003 |
| *TMEM154* | *transmembrane protein 154* | | 1.19 | 0.028 | 1.23 | 0.003 |
| *ZNF521* | *zinc finger protein 521* | | 1.14 | 0.054 | 1.23 | 0.012 |
| *ITGB4* | *integrin, beta 4* | | 1.23 | 0.008 | 1.23 | 0.043 |
| *AHNAK* | *AHNAK nucleoprotein* | | -1.00 | 0.987 | 1.22 | 0.006 |
| *TFPI* | *tissue factor pathway inhibitor (lipoprotein-associated coagulation inhibitor)* | | 1.24 | 0.004 | 1.22 | 0.023 |
| *MS4A2* | *membrane-spanning 4-domains, subfamily A, member 2* | | 1.21 | 0.095 | 1.22 | 0.037 |
| *TMOD2* | *tropomodulin 2 (neuronal)* | | 1.06 | 0.466 | 1.22 | 0.017 |
| *TNC* | *tenascin C* | | 1.14 | 0.211 | 1.22 | 0.023 |
| *SKOR1* | *SKI family transcriptional corepressor 1* | | 1.21 | 0.003 | 1.22 | 0.012 |
| *LOC100737797* | *elongation factor 1-alpha, oocyte form-like* | | -1.08 | 0.447 | 1.22 | 0.049 |
| *RIPPLY1* | *ripply transcriptional repressor 1* | | 1.18 | 0.117 | 1.22 | 0.029 |
| *MCTP1* | *multiple C2 domains, transmembrane 1* | | 1.03 | 0.685 | 1.22 | 0.012 |
| *SSTR1* | *somatostatin receptor 1* | | 1.13 | 0.149 | 1.22 | 0.028 |
| *ACVR1* | *activin A receptor, type I* | | 1.12 | 0.100 | 1.21 | 0.001 |
| *LOC102157584* | *calmodulin-like* | | 1.00 | 0.959 | 1.21 | 0.044 |
| *CLDN5* | *claudin 5* | | 1.22 | 0.001 | 1.21 | 0.001 |
| *TTC25* | *tetratricopeptide repeat domain 25* | | 1.10 | 0.053 | 1.21 | 0.020 |
| *IQCK* | *IQ motif containing K* | | 1.10 | 0.239 | 1.21 | 0.044 |
| *SYNPO2* | *synaptopodin 2* | | 1.14 | 0.372 | 1.21 | 0.030 |
| *LOC100157955* | *olfactory receptor 4K13-like* | | 1.28 | 0.018 | 1.21 | 0.005 |
| *AFF2* | *AF4/FMR2 family, member 2* | | 1.23 | 0.003 | 1.21 | 0.006 |
| *BTBD1* | *BTB (POZ) domain containing 1* | | 1.11 | 0.154 | 1.21 | 0.006 |
| *C14H10orf128* | *chromosome 14 open reading frame, human C10orf128* | | 1.06 | 0.432 | 1.21 | 0.006 |
| *PCGF5* | *polycomb group ring finger 5* | | 1.10 | 0.252 | 1.21 | 0.043 |
| *NDUFA1* | *NADH dehydrogenase (ubiquinone) 1 alpha subcomplex, 1, 7.5kDa* | | 1.14 | 0.196 | 1.21 | 0.001 |
| *LOC100738565* | *kinesin-like protein KIF17* | | 1.12 | 0.260 | 1.21 | 0.025 |
|  |  | |  |  |  |  |
| *Down-regulated* |  | |  |  |  |  |
| *TMEM52B* | | transmembrane protein 52B | -4.40 | 0.001 | -3.44 | 0.019 |
| *ADAD1* | | adenosine deaminase domain containing 1 (testis-specific) | -1.67 | 0.192 | -2.78 | 0.026 |
| *GTSF1* | | gametocyte specific factor 1 | -1.45 | 0.110 | -2.08 | 0.036 |
| *FKBP1B* | | FK506 binding protein 1B, 12.6 kDa | -1.06 | 0.710 | -1.53 | 0.013 |
| *LOC100525902* | | calcium/calmodulin-dependent 3,5-cyclic nucleotide phosphodiesterase 1C | -1.12 | 0.542 | -1.52 | 0.028 |
| *GPRIN3* | | GPRIN family member 3 | -1.25 | 0.099 | -1.38 | 0.003 |
| *LOC100523488* | | olfactory receptor 140-like | -1.22 | 0.051 | -1.37 | 0.001 |
| *LOC100524036* | | sortilin-related receptor | -1.24 | 0.094 | -1.37 | 0.026 |
| *LOC100154120* | | olfactory receptor 2G6-like | -1.09 | 0.541 | -1.36 | 0.003 |
| *SLPI* | | secretory leukocyte peptidase inhibitor | -1.30 | 0.042 | -1.34 | 0.001 |
| *SLPI* | | secretory leukocyte peptidase inhibitor | -1.30 | 0.042 | -1.34 | 0.001 |
| *LOC100516876* | | olfactory receptor 1A1-like | -1.22 | 0.251 | -1.34 | 0.009 |
| *CLEC4F* | | C-type lectin domain family 4, member F | 1.04 | 0.644 | -1.34 | 0.036 |
| *CCL14* | | chemokine (C-C motif) ligand 14 | -1.11 | 0.387 | -1.33 | 0.016 |
| *LOC100739773* | | alpha-fetoprotein-like | -1.37 | 0.055 | -1.33 | 0.047 |
| *LOC102165749* | | vomeronasal type-2 receptor 116-like | 1.09 | 0.309 | -1.32 | 0.019 |
| *ASS1* | | argininosuccinate synthase 1 | -1.09 | 0.408 | -1.32 | 0.016 |
| *CYP26A1* | | cytochrome P450, family 26, subfamily A, polypeptide 1 | -1.04 | 0.660 | -1.32 | 0.011 |
| *URB2* | | URB2 ribosome biogenesis 2 homolog (S. cerevisiae) | -1.11 | 0.345 | -1.31 | 0.003 |
| *ARG2* | | arginase 2 | -1.05 | 0.752 | -1.30 | 0.030 |
| *PRC1* | | protein regulator of cytokinesis 1 | -1.12 | 0.372 | -1.30 | 0.020 |
| *LOC106510157* | | uncharacterized LOC106510157 | -1.24 | 0.104 | -1.30 | 0.039 |
| *MLX* | | MLX, MAX dimerization protein | -1.08 | 0.253 | -1.29 | 0.007 |
| *LOC102167096* | | immunoglobulin lambda-like polypeptide 1 | 1.10 | 0.585 | -1.29 | 0.038 |
| *LOC100626407* | | cationic amino acid transporter 3-like | -1.21 | 0.056 | -1.28 | 0.004 |
| *CTXN2* | | cortexin 2 | -1.07 | 0.278 | -1.28 | 0.011 |
| *LOC100516208* | | olfactory receptor 4F6-like | -1.09 | 0.358 | -1.28 | 0.014 |
| *LOC102159844* | | melanoma-associated antigen B4-like | -1.16 | 0.213 | -1.28 | 0.002 |
| *DNAJB9* | | DnaJ (Hsp40) homolog, subfamily B, member 9 | -1.29 | 0.033 | -1.27 | 0.023 |
| *SLAMF1* | | signaling lymphocytic activation molecule family member 1 | -1.17 | 0.025 | -1.27 | 0.007 |
| *FAM19A1* | | family with sequence similarity 19 (chemokine (C-C motif)-like), member A1 | -1.14 | 0.328 | -1.26 | 0.016 |
| *SLC25A33* | | solute carrier family 25 (pyrimidine nucleotide carrier), member 33 | -1.03 | 0.773 | -1.26 | 0.026 |
| *MIR935* | | microRNA mir-935 | 1.03 | 0.689 | -1.26 | 0.027 |
| *FANCF* | | Fanconi anemia, complementation group F | -1.12 | 0.200 | -1.26 | 0.004 |
| *CCL4* | | chemokine (C-C motif) ligand 4 | -1.08 | 0.283 | -1.25 | 0.005 |
| *LOC100739121* | | uncharacterized LOC100739121 | -1.02 | 0.894 | -1.25 | 0.023 |
| *LOC106504070* | | arginase-1-like | 1.04 | 0.763 | -1.25 | 0.030 |
| *CD5* | | CD5 molecule | -1.13 | 0.278 | -1.25 | 0.007 |
| *NFIL3* | | nuclear factor, interleukin 3 regulated | -1.16 | 0.173 | -1.25 | 0.010 |
| *IL17D* | | interleukin 17D | -1.07 | 0.377 | -1.24 | 0.009 |
| *LOC100737167* | | peptidyl-prolyl cis-trans isomerase FKBP5-like | -1.06 | 0.753 | -1.24 | 0.003 |
| *HSPH1* | | heat shock 105kDa/110kDa protein 1 | -1.07 | 0.591 | -1.24 | 0.038 |
| *AVEN* | | apoptosis, caspase activation inhibitor | -1.33 | 0.005 | -1.24 | 0.033 |
| *ZPR1* | | ZPR1 zinc finger | -1.11 | 0.370 | -1.24 | 0.004 |
| *LOC100624136* | | ankyrin repeat domain-containing protein 45 | -1.01 | 0.883 | -1.23 | 0.018 |
| *TMEM101* | | transmembrane protein 101 | -1.09 | 0.217 | -1.23 | 0.006 |
| *LOC100526097* | | olfactory receptor-like protein OLF4 | -1.01 | 0.872 | -1.23 | 0.049 |
| *C3H2orf44* | | chromosome 3 open reading frame, human C2orf44 | 1.01 | 0.890 | -1.23 | 0.029 |
| *EOGT* | | EGF domain-specific O-linked N-acetylglucosamine (GlcNAc) transferase | -1.16 | 0.148 | -1.23 | 0.018 |
| *HSD3B1* | | hydroxy-delta-5-steroid dehydrogenase, 3 beta- and steroid delta-isomerase 1 | -1.19 | 0.053 | -1.23 | 0.042 |
| *LOC100622976* | | forkhead box protein F2 | -1.02 | 0.842 | -1.22 | 0.045 |
| *LOC100514488* | | olfactory receptor 3A2 | -1.04 | 0.708 | -1.22 | 0.042 |
| *LOC100626772* | | zinc finger protein 142-like | -1.10 | 0.227 | -1.22 | 0.022 |
| *NDUFAF4* | | NADH dehydrogenase (ubiquinone) complex I, assembly factor 4 | -1.03 | 0.803 | -1.22 | 0.031 |
| *ZFAND2A* | | zinc finger, AN1-type domain 2A | -1.06 | 0.608 | -1.22 | 0.019 |
| *GABRA1* | | gamma-aminobutyric acid (GABA) A receptor, alpha 1 | -1.11 | 0.374 | -1.22 | 0.031 |
| *LACC1* | | laccase (multicopper oxidoreductase) domain containing 1 | -1.26 | 0.008 | -1.21 | 0.024 |
| *LOC100154916* | | histone H4 | -1.17 | 0.034 | -1.21 | 0.035 |
| *LOC106506220* | | uncharacterized LOC106506220 | -1.04 | 0.359 | -1.21 | 0.007 |
| *RPF2* | | ribosome production factor 2 homolog | -1.18 | 0.067 | -1.21 | 0.012 |
| *TTPAL* | | tocopherol (alpha) transfer protein-like | 1.04 | 0.673 | -1.21 | 0.002 |

*Filter criteria: FC > 1.2 and < -1.2 and *P* < 0.05 between groups IM10 vs. CON. The FCs for these genes and *P*-values are also shown for the comparison between groups IM5 vs. CON. FCs were calculated from the signal log ratios, which were calculated from *n* = 6 microarrays per group.

Abbreviations: CON, control group; FC, fold change; IM5, insect meal 5% group; IM10, insect meal 10% group.

**Table S5**

qPCR validation of microarray data for selected differentially expressed transcripts (FC > 1.2 or < -1.2, *P* < 0.05) in the liver of pigs of group IM10 compared with group CON.*

|  | FC  IM10 vs. CON | | *P*-value |
| --- | --- | --- | --- |
| Gene symbol | Microarray | qPCR | qPCR |
| *CISH* | 3.45 | 9.85 | < 0.05 |
| *HAMP* | 2.97 | 2.50 | < 0.05 |
| *ACTG2* | 2.87 | 1.71 | < 0.05 |
| *ID1* | 1.60 | 1.23 | n.s. |
| *FCER1A* | 1.52 | 1.17 | n.s. |
| *TFRC* | 1.45 | 1.18 | n.s. |
| *CUEDC1* | 1.42 | -1.05 | n.s. |
| *NOV* | 1.41 | 1.35 | n.s. |
| *SMAD7* | 1.37 | 1.18 | n.s. |
| *TAGLN* | 1.34 | 1.63 | < 0.05 |
| *ADAD1* | -2.78 | -1.67 | < 0.05 |
| *GTSF1* | -2.08 | -1.98 | < 0.05 |
| *APCS* | -1.89 | -1.94 | < 0.05 |
| *DUSP6* | -1.57 | -1.57 | < 0.05 |
| *GPRIN3* | -1.38 | -1.35 | < 0.05 |
| *SLC6A8* | -1.36 | -1.30 | n.s. |
| *CLEC4F* | -1.34 | -1.34 | n.s. |
| *SLPI* | -1.34 | -1.63 | < 0.05 |
| *CCL14* | -1.33 | -1.32 | n.s. |
| *CYP26A1* | -1.32 | -1.70 | < 0.05 |

The microarray FC was calculated from the signal log ratios as follows: 2^Signal log ratio^, if signal log ratio ≥ 0 and (-1) x 2^–(Signal log ratio)^, if signal log ratio < 0. Signal log ratios were calculated from *n* = 5 microarrays per group. The qPCR FC was calculated analogously from normalized 2^-ΔCt^ ratios. Normalized 2^-ΔCt^ expression was calculated from *n* = 10 samples per group.

Abbreviations: CON, control group; FC, fold change; IM10, insect meal 10% group.

**Table S6**

Up- and down-regulated genes in the gastrocnemius muscle of pigs of group IM10 compared with group CON.*

| Gene symbol | Gene description | IM5 vs. CON | | IM10 vs. CON | |
| --- | --- | --- | --- | --- | --- |
|  |  | FC | *P*-value | FC | *P*-value |
| *Up-regulated* |  |  |  |  |  |
| *RDH16/LOC100512656* | retinol dehydrogenase 16 | 4.15 | 0.032 | 5.09 | 0.010 |
| *MIR29B-2* | microRNA mir-29b-2 | 1.67 | 0.003 | 1.87 | 0.000 |
| *VAV1* | vav 1 guanine nucleotide exchange factor | 1.11 | 0.390 | 1.57 | 0.046 |
| *ATF5* | activating transcription factor 5 | -1.05 | 0.732 | 1.51 | 0.034 |
| *LOC100515430* | olfactory receptor 4S1 | 1.21 | 0.095 | 1.48 | 0.002 |
| *HGF* | hepatocyte growth factor (hepapoietin A; scatter factor) | 1.55 | 0.001 | 1.46 | 0.035 |
| *LOC100626456* | olfactory receptor 7A10-like | 1.04 | 0.593 | 1.45 | 0.001 |
| *LOC100620475* | putative protein ZNF720 | 1.49 | 0.011 | 1.44 | 0.018 |
| *LOC100514264* | RNA-binding protein 3 pseudogene | 1.01 | 0.931 | 1.44 | 0.029 |
| *LOC100514264* | RNA-binding protein 3 pseudogene | 1.01 | 0.931 | 1.44 | 0.029 |
| *LOC100514940* | melanoma-associated antigen 10-like | 1.34 | 0.043 | 1.43 | 0.040 |
| *MIR29C* | microRNA mir-29c | 1.51 | 0.005 | 1.40 | 0.008 |
| *LOC100524754* | olfactory receptor 52B4-like | 1.28 | 0.055 | 1.39 | 0.004 |
| *CLK1* | CDC-like kinase 1 | 1.26 | 0.014 | 1.37 | 0.001 |
| *OCIAD2* | OCIA domain containing 2 | 1.19 | 0.270 | 1.37 | 0.031 |
| *LOC100739867* | myoferlin-like | 1.18 | 0.036 | 1.36 | 0.037 |
| *LOC100624864* | acetylcholine receptor subunit epsilon-like | 1.38 | 0.039 | 1.36 | 0.049 |
| *OLFM3* | olfactomedin 3 | 1.04 | 0.825 | 1.36 | 0.044 |
| *LOC100512543* | olfactory receptor 2W1-like | 1.47 | 0.003 | 1.34 | 0.023 |
| *LOC106504915* | olfactory receptor 151-like | 1.10 | 0.213 | 1.34 | 0.042 |
| *GBE1* | glucan (1,4-alpha-), branching enzyme 1 | 1.31 | 0.077 | 1.34 | 0.038 |
| *SOX2* | SRY (sex determining region Y)-box 2 | 1.14 | 0.082 | 1.33 | 0.004 |
| *BEST3* | bestrophin 3 | 1.28 | 0.084 | 1.33 | 0.023 |
| *LOC100522598* | 40S ribosomal protein S15a pseudogene | 1.05 | 0.536 | 1.33 | 0.013 |
| *KCNJ2* | potassium channel, inwardly rectifying subfamily J, member 2 | 1.09 | 0.284 | 1.32 | 0.006 |
| *LOC100516511* | olfactory receptor 1A1 | 1.13 | 0.276 | 1.32 | 0.017 |
| *RBP7* | retinol binding protein 7, cellular | 1.33 | 0.052 | 1.31 | 0.045 |
| *STXBP2* | syntaxin binding protein 2 | 1.17 | 0.010 | 1.31 | 0.002 |
| *LOC100152126* | zinc finger and SCAN domain-containing protein 12 | 1.18 | 0.094 | 1.31 | 0.004 |
| *LOC100514912* | elongation factor 1-alpha, oocyte form-like | 1.10 | 0.281 | 1.30 | 0.022 |
| *CTXN3* | cortexin 3 | 1.86 | 0.027 | 1.30 | 0.004 |
| *EXOSC6* | exosome component 6 | 1.17 | 0.112 | 1.30 | 0.037 |
| *TPBG* | trophoblast glycoprotein | 1.26 | 0.026 | 1.29 | 0.034 |
| *LOC100514709* | small ubiquitin-related modifier 1 pseudogene | 1.08 | 0.433 | 1.29 | 0.021 |
| *STEAP4* | STEAP family member 4 | 1.06 | 0.531 | 1.29 | 0.008 |
| *LOC100515302* | histone H2B type 1-L-like | 1.04 | 0.670 | 1.29 | 0.029 |
| *CLCA1* | chloride channel accessory 1 | 1.15 | 0.109 | 1.29 | 0.012 |
| *CXCR4* | chemokine (C-X-C motif) receptor 4 | 1.23 | 0.027 | 1.29 | 0.004 |
| *LOC102158682* | olfactory receptor 52D1-like | 1.06 | 0.578 | 1.28 | 0.025 |
| *HSF5* | heat shock transcription factor family member 5 | 1.28 | 0.025 | 1.28 | 0.003 |
| *IVNS1ABP* | influenza virus NS1A binding protein | 1.06 | 0.525 | 1.27 | 0.034 |
| *LOC100620754* | muscle-related coiled-coil protein | 1.39 | 0.007 | 1.27 | 0.031 |
| *LOC100736752* | palmitoyltransferase ZDHHC13-like | 1.19 | 0.135 | 1.27 | 0.035 |
| *LOC100519264* | EF-hand calcium-binding domain-containing protein 1-like | 1.15 | 0.260 | 1.27 | 0.036 |
| *LOC102159415* | neuronal tyrosine-phosphorylated phosphoinositide-3-kinase adapter 2-like | 1.07 | 0.406 | 1.27 | 0.039 |
| *PADI4* | peptidyl arginine deiminase, type IV | 1.23 | 0.009 | 1.27 | 0.001 |
| *LOC100154118* | olfactory receptor 10C1-like | -1.01 | 0.895 | 1.27 | 0.029 |
| *HK2* | hexokinase 2 | 1.25 | 0.138 | 1.27 | 0.038 |
| *LOC100156011* | glutaredoxin-1 pseudogene | -1.02 | 0.836 | 1.27 | 0.013 |
| *MYL4* | myosin, light chain 4, alkali; atrial, embryonic | 1.07 | 0.613 | 1.27 | 0.011 |
| *LOC100738683* | olfactory receptor 2D3-like | 1.06 | 0.717 | 1.26 | 0.042 |
| *LRIG3* | leucine-rich repeats and immunoglobulin-like domains 3 | 1.22 | 0.031 | 1.26 | 0.011 |
| *FMR1NB* | fragile X mental retardation 1 neighbor | 1.10 | 0.216 | 1.26 | 0.002 |
| *LOC100518088* | uncharacterized LOC100518088 | 1.13 | 0.210 | 1.26 | 0.048 |
| *LOC100523809* | olfactory receptor 52N4-like | 1.15 | 0.302 | 1.26 | 0.050 |
| *TUBB2B* | tubulin, beta 2B class IIb | 1.09 | 0.447 | 1.26 | 0.023 |
| *LPAR6* | lysophosphatidic acid receptor 6 | 1.10 | 0.367 | 1.26 | 0.032 |
| *LOC100517850* | multidrug resistance-associated protein 4-like | 1.06 | 0.666 | 1.25 | 0.046 |
| *GNAI3* | guanine nucleotide binding protein (G protein), alpha inhibiting activity polypeptide 3 | 1.16 | 0.163 | 1.25 | 0.047 |
| *LRTM2* | leucine-rich repeats and transmembrane domains 2 | -1.02 | 0.749 | 1.25 | 0.025 |
| *IFN-ALPHA-17* | interferon-alpha-17 | 1.08 | 0.526 | 1.25 | 0.026 |
| *LOC102163521* | nuclease-sensitive element-binding protein 1 pseudogene | -1.03 | 0.608 | 1.25 | 0.001 |
| *MTBP* | MDM2 binding protein | 1.07 | 0.430 | 1.24 | 0.043 |
| *PACS1* | phosphofurin acidic cluster sorting protein 1 | 1.12 | 0.039 | 1.24 | 0.001 |
| *HBZ* | hemoglobin, zeta | 1.07 | 0.325 | 1.24 | 0.025 |
| *MAP3K1* | mitogen-activated protein kinase kinase kinase 1, E3 ubiquitin protein ligase | 1.13 | 0.163 | 1.24 | 0.021 |
| *MDP1* | magnesium-dependent phosphatase 1 | 1.02 | 0.709 | 1.24 | 0.004 |
| *PLD4* | phospholipase D family, member 4 | -1.04 | 0.594 | 1.24 | 0.037 |
| *S1PR3* | sphingosine-1-phosphate receptor 3 | 1.15 | 0.048 | 1.24 | 0.007 |
| *TMEM163* | transmembrane protein 163 | 1.14 | 0.276 | 1.23 | 0.003 |
| *AIG1* | androgen-induced 1 | 1.30 | 0.031 | 1.23 | 0.033 |
| *CD5L* | CD5 molecule-like | 1.19 | 0.046 | 1.23 | 0.034 |
| *LOC102164035* | pleckstrin homology domain-containing family A member 5-like | 1.24 | 0.037 | 1.23 | 0.035 |
| *LOC100521308* | DDB1- and CUL4-associated factor 12-like protein 2 | -1.01 | 0.901 | 1.22 | 0.014 |
| *LOC396625* | glutathione reductase | 1.15 | 0.045 | 1.22 | 0.004 |
| *LOC100523557* | interferon-inducible GTPase 5-like | 1.04 | 0.560 | 1.22 | 0.030 |
| *PDIA6* | protein disulfide isomerase family A, member 6 | 1.05 | 0.331 | 1.22 | 0.046 |
| *LOC102162711* | zinc finger protein 14 homolog | 1.19 | 0.016 | 1.21 | 0.018 |
| *SHPK* | sedoheptulokinase | -1.02 | 0.803 | 1.21 | 0.030 |
| *C3H2orf78* | chromosome 3 open reading frame, human C2orf78 | 1.09 | 0.495 | 1.21 | 0.010 |
| *LOC100511430* | spermatid nuclear transition protein 4 | 1.20 | 0.041 | 1.21 | 0.015 |
| *C2CD5* | C2 calcium-dependent domain containing 5 | 1.18 | 0.076 | 1.21 | 0.015 |
| *BMPER* | BMP binding endothelial regulator | 1.14 | 0.043 | 1.21 | 0.028 |
| *RPL26L1* | ribosomal protein L26-like 1 | 1.10 | 0.272 | 1.21 | 0.026 |
| *LOC100521466* | olfactory receptor 52L1 | 1.01 | 0.890 | 1.21 | 0.015 |
| *FAM110B* | family with sequence similarity 110, member B | 1.13 | 0.060 | 1.21 | 0.011 |
| *LOC106504191* | olfactory receptor 1J4 | 1.09 | 0.431 | 1.21 | 0.049 |
|  |  |  |  |  |  |
| *Down-regulated* |  |  |  |  |  |
| *BTG2* | BTG family, member 2 | -2.14 | 0.015 | -3.63 | 0.000 |
| *ATF3* | activating transcription factor 3 | -2.25 | 0.042 | -2.41 | 0.006 |
| *ABRA* | actin binding Rho activating protein | -1.49 | 0.022 | -1.99 | 0.003 |
| *NMNAT1* | nicotinamide nucleotide adenylyltransferase 1 | -1.34 | 0.103 | -1.91 | 0.002 |
| *MIR98* | microRNA mir-98 | -1.49 | 0.099 | -1.82 | 0.002 |
| *ARG2* | arginase 2 | -1.76 | 0.131 | -1.76 | 0.046 |
| *JUN* | jun proto-oncogene | -1.25 | 0.341 | -1.75 | 0.021 |
| *ENHO* | energy homeostasis associated | -1.34 | 0.377 | -1.69 | 0.003 |
| *PFKFB3* | 6-phosphofructo-2-kinase/fructose-2,6-biphosphatase 3 | -1.41 | 0.040 | -1.66 | 0.001 |
| *IFRD1* | interferon-related developmental regulator 1 | -1.41 | 0.064 | -1.62 | 0.021 |
| *PFKFB3* | 6-phosphofructo-2-kinase/fructose-2,6-biphosphatase 3 | -1.37 | 0.088 | -1.59 | 0.006 |
| *PDZD9* | PDZ domain containing 9 | -1.34 | 0.153 | -1.58 | 0.031 |
| *GPA33* | glycoprotein A33 (transmembrane) | -1.41 | 0.041 | -1.57 | 0.010 |
| *LOC100152127* | putative olfactory receptor 2B8 | -1.39 | 0.111 | -1.55 | 0.012 |
| *AQP3* | aquaporin 3 (Gill blood group) | -1.38 | 0.375 | -1.49 | 0.020 |
| *RXRG* | retinoid X receptor, gamma | -1.15 | 0.544 | -1.48 | 0.039 |
| *VGLL3* | vestigial-like family member 3 | -1.26 | 0.038 | -1.42 | 0.003 |
| *LOC100153719* | putative olfactory receptor 2B8 | -1.05 | 0.705 | -1.42 | 0.011 |
| *CHST9* | carbohydrate (N-acetylgalactosamine 4-0) sulfotransferase 9 | -1.27 | 0.196 | -1.41 | 0.029 |
| *PBX3* | pre-B-cell leukemia homeobox 3 | -1.24 | 0.225 | -1.40 | 0.044 |
| *LOC100524133* | olfactory receptor 18-like | -1.27 | 0.163 | -1.40 | 0.042 |
| *PER1* | period circadian clock 1 | -1.05 | 0.712 | -1.39 | 0.002 |
| *CXCL11* | chemokine (C-X-C motif) ligand 11 | -1.43 | 0.002 | -1.39 | 0.015 |
| *FGF6* | fibroblast growth factor 6 | -1.58 | 0.029 | -1.39 | 0.050 |
| *SLC29A2* | solute carrier family 29 (equilibrative nucleoside transporter), member 2 | -1.24 | 0.052 | -1.38 | 0.004 |
| *EFCAB8* | EF-hand calcium binding domain 8 | -1.35 | 0.024 | -1.37 | 0.011 |
| *LOC100621968* | aurora kinase A-like | -1.11 | 0.382 | -1.37 | 0.013 |
| *TMEM109* | transmembrane protein 109 | -1.10 | 0.502 | -1.36 | 0.017 |
| *LOC100516421* | olfactory receptor 2G3 | -1.04 | 0.764 | -1.36 | 0.006 |
| *DNAJB5* | DnaJ (Hsp40) homolog, subfamily B, member 5 | -1.16 | 0.200 | -1.35 | 0.004 |
| *LOC102163654* | autophagy-related protein 2 homolog B-like | 1.03 | 0.857 | -1.34 | 0.019 |
| *CARNS1* | carnosine synthase 1 | -1.15 | 0.405 | -1.34 | 0.020 |
| *LOC100620963* | T-complex protein 11-like protein 2 | -1.20 | 0.199 | -1.34 | 0.045 |
| *LOC102159988* | forkhead box protein O1-like | -1.11 | 0.046 | -1.33 | 0.004 |
| *CLVS2* | clavesin 2 | -1.12 | 0.269 | -1.31 | 0.014 |
| *LOC100153886* | olfactory receptor 11H6-like | -1.18 | 0.077 | -1.31 | 0.009 |
| *APAF1* | apoptotic peptidase activating factor 1 | -1.34 | 0.004 | -1.30 | 0.034 |
| *GRM4* | glutamate receptor, metabotropic 4 | -1.19 | 0.073 | -1.30 | 0.012 |
| *GDAP1* | ganglioside induced differentiation associated protein 1 | -1.01 | 0.932 | -1.29 | 0.010 |
| *LOC100516070* | olfactory receptor 2T29-like | -1.14 | 0.272 | -1.29 | 0.028 |
| *ECHDC3* | enoyl CoA hydratase domain containing 3 | -1.14 | 0.184 | -1.29 | 0.008 |
| *LOC102162678* | olfactory receptor 6C1-like | -1.07 | 0.364 | -1.29 | 0.007 |
| *LOC100737069* | olfactory receptor 8S1-like | -1.22 | 0.028 | -1.28 | 0.017 |
| *LOC100156990* | olfactory receptor 8K5-like | -1.13 | 0.149 | -1.28 | 0.021 |
| *ANGPTL4* | angiopoietin-like 4 | -1.29 | 0.025 | -1.28 | 0.027 |
| *LOC100519942* | olfactory receptor 9G4-like | -1.22 | 0.155 | -1.28 | 0.025 |
| *MIR199A-1* | microRNA mir-199a-1 | 1.06 | 0.614 | -1.28 | 0.035 |
| *CNP* | 2,3-cyclic nucleotide 3 phosphodiesterase | -1.23 | 0.020 | -1.27 | 0.003 |
| *LOC102167096* | immunoglobulin lambda-like polypeptide 1 | -1.33 | 0.092 | -1.27 | 0.048 |
| *DYRK3* | dual-specificity tyrosine-(Y)-phosphorylation regulated kinase 3 | 1.05 | 0.599 | -1.27 | 0.012 |
| *LOC100521545* | olfactory receptor-like protein OLF4 | -1.07 | 0.541 | -1.27 | 0.029 |
| *LOC100156762* | olfactory receptor 6C4-like | -1.20 | 0.076 | -1.27 | 0.032 |
| *CACNB3* | calcium channel, voltage-dependent, beta 3 subunit | -1.13 | 0.101 | -1.26 | 0.014 |
| *PPT2* | palmitoyl-protein thioesterase 2 | -1.13 | 0.153 | -1.26 | 0.028 |
| *XPR1* | xenotropic and polytropic retrovirus receptor 1 | -1.15 | 0.116 | -1.26 | 0.031 |
| *LOC102163994* | VWFA and cache domain-containing protein 1-like | 1.00 | 0.965 | -1.26 | 0.040 |
| *LOC100155532* | olfactory receptor 49-like | -1.08 | 0.290 | -1.26 | 0.000 |
| *MIR204* | microRNA mir-204 | -1.25 | 0.004 | -1.26 | 0.021 |
| *LOC100522138* | protein FAM65A | -1.05 | 0.519 | -1.26 | 0.001 |
| *STARD4* | StAR-related lipid transfer (START) domain containing 4 | -1.13 | 0.318 | -1.25 | 0.038 |
| *ANKRD13B* | ankyrin repeat domain 13B | -1.04 | 0.613 | -1.25 | 0.045 |
| *MVB12B* | multivesicular body subunit 12B | -1.03 | 0.729 | -1.25 | 0.001 |
| *APTX* | aprataxin | -1.22 | 0.057 | -1.25 | 0.007 |
| *DMC1* | DNA meiotic recombinase 1 | -1.04 | 0.685 | -1.25 | 0.018 |
| *RAB3A* | RAB3A, member RAS oncogene family | -1.14 | 0.172 | -1.25 | 0.036 |
| *GIF* | gastric intrinsic factor (vitamin B synthesis) | -1.10 | 0.280 | -1.25 | 0.025 |
| *LOC100511363* | olfactory receptor 1J4-like | -1.00 | 0.998 | -1.25 | 0.022 |
| *C10H1orf53* | chromosome 10 open reading frame, human C1orf53 | 1.03 | 0.778 | -1.25 | 0.026 |
| *LOC102166466* | hemicentin-1-like | -1.12 | 0.292 | -1.25 | 0.010 |
| *ZC3H12B* | zinc finger CCCH-type containing 12B | -1.17 | 0.154 | -1.25 | 0.012 |
| *LOC100156766* | olfactory receptor 4F3/4F16/4F29 | -1.22 | 0.030 | -1.24 | 0.015 |
| *PNCK* | pregnancy up-regulated nonubiquitous CaM kinase | -1.34 | 0.007 | -1.24 | 0.019 |
| *CC2D2B* | coiled-coil and C2 domain containing 2B | -1.10 | 0.126 | -1.24 | 0.000 |
| *LOC106508607* | uncharacterized LOC106508607 | -1.11 | 0.243 | -1.24 | 0.023 |
| *MLXIP* | MLX interacting protein | -1.06 | 0.507 | -1.23 | 0.005 |
| *LOC100737500* | DNA polymerase eta-like | 1.07 | 0.656 | -1.23 | 0.039 |
| *LOC100156159* | olfactory receptor 5M3 | -1.20 | 0.074 | -1.23 | 0.023 |
| *LOC100157058* | filamin A-interacting protein 1-like | -1.13 | 0.036 | -1.23 | 0.005 |
| *FBXW8* | F-box and WD repeat domain containing 8 | -1.11 | 0.313 | -1.23 | 0.044 |
| *CHCHD5* | coiled-coil-helix-coiled-coil-helix domain containing 5 | -1.05 | 0.616 | -1.23 | 0.004 |
| *AQP11* | aquaporin 11 | -1.22 | 0.137 | -1.22 | 0.025 |
| *CCNE1* | cyclin E1 | -1.07 | 0.580 | -1.22 | 0.031 |
| *LOC100737213* | dual specificity mitogen-activated protein kinase kinase 2-like | -1.12 | 0.118 | -1.22 | 0.003 |
| *LOC106506567* | uncharacterized LOC106506567 | -1.02 | 0.824 | -1.22 | 0.049 |
| *NPS* | neuropeptide S | -1.10 | 0.306 | -1.22 | 0.011 |
| *NPS* | neuropeptide S | -1.10 | 0.306 | -1.22 | 0.011 |
| *PLCE1* | phospholipase C, epsilon 1 | 1.11 | 0.207 | -1.22 | 0.041 |
| *LOC100524934* | DNA-directed RNA polymerases I, II, and III subunit RPABC2 | -1.07 | 0.360 | -1.22 | 0.020 |
| *SNX19* | sorting nexin 19 | -1.07 | 0.351 | -1.22 | 0.015 |
| *GPR153* | G protein-coupled receptor 153 | -1.10 | 0.154 | -1.22 | 0.028 |
| *LOC100523609* | cationic amino acid transporter 3-like | 1.04 | 0.480 | -1.22 | 0.038 |
| *SCAMP3* | secretory carrier membrane protein 3 | -1.10 | 0.170 | -1.21 | 0.028 |
| *FUT1* | fucosyltransferase 1 (galactoside 2-alpha-L-fucosyltransferase, H blood group) | -1.10 | 0.269 | -1.21 | 0.024 |
| *CLMP* | CXADR-like membrane protein | -1.15 | 0.110 | -1.21 | 0.017 |
| *LOC102162240* | tubulin polyglutamylase TTLL4 | -1.05 | 0.621 | -1.21 | 0.006 |
| *BNIPL* | BCL2/adenovirus E1B 19kD interacting protein like | -1.23 | 0.025 | -1.21 | 0.008 |
| *MCL1* | myeloid cell leukemia 1 | -1.06 | 0.479 | -1.21 | 0.037 |
| *ABCB9* | ATP-binding cassette, sub-family B (MDR/TAP), member 9 | -1.18 | 0.025 | -1.21 | 0.027 |
| *LOC100511652* | glycerophosphodiester phosphodiesterase domain-containing protein 4 | -1.07 | 0.331 | -1.21 | 0.048 |
| *POC5* | POC5 centriolar protein | 1.01 | 0.925 | -1.21 | 0.010 |
| *NR4A2* | nuclear receptor subfamily 4, group A, member 2 | 1.00 | 0.994 | -1.21 | 0.032 |
| *FGF9* | fibroblast growth factor 9 | -1.08 | 0.364 | -1.21 | 0.038 |
| *RSPRY1* | ring finger and SPRY domain containing 1 | -1.08 | 0.425 | -1.21 | 0.025 |
| *SMAD6* | SMAD family member 6 | -1.07 | 0.311 | -1.21 | 0.012 |
| *PPM1J* | protein phosphatase, Mg2+/Mn2+ dependent, 1J | -1.09 | 0.094 | -1.21 | 0.000 |
| *APOA1BP* | apolipoprotein A-I binding protein | -1.02 | 0.797 | -1.21 | 0.010 |
| *LOC100157354* | interferon omega 5 | 1.00 | 0.995 | -1.21 | 0.045 |
| *NRXN2* | neurexin 2 | -1.09 | 0.227 | -1.21 | 0.040 |
| *TMEM136* | transmembrane protein 136 | -1.05 | 0.477 | -1.21 | 0.030 |
| *MIR190A* | microRNA mir-190a | -1.03 | 0.704 | -1.21 | 0.049 |
| *LOC100519868* | V-type proton ATPase 116 kDa subunit a isoform 1-like | -1.04 | 0.621 | -1.21 | 0.042 |

*Filter criteria: FC > 1.2 and < -1.2 and *P* < 0.05 between groups IM10 vs. CON. The FCs for these genes and *P*-values are also shown for the comparison between groups IM5 vs. CON. FCs were calculated from the signal log ratios, which were calculated from *n* = 6 microarrays per group.

Abbreviations: CON, control group; FC, fold change; IM5, insect meal 5% group; IM10, insect meal 10% group.

**Table S7**

qPCR validation of microarray data for selected differentially expressed transcripts (FC > 1.2 or < -1.2, *P* < 0.05) in gastrocnemius muscle of pigs of group IM10 compared with group CON.*

|  | FC  IM10 vs. CON | | *P*-value |
| --- | --- | --- | --- |
| Gene symbol | Microarray | qPCR | qPCR |
| *LOC100512656* | 5.09 | 2.29 | < 0.05 |
| *VAV1* | 1.57 | 1.66 | n.s. |
| *ATF5* | 1.51 | 1.93 | < 0.05 |
| *CLK1* | 1.37 | 1.15 | n.s. |
| *OCIAD2* | 1.37 | 1.15 | n.s. |
| *OLFM3* | 1.36 | 1.62 | < 0.05 |
| *GBE1* | 1.34 | 1.00 | n.s. |
| *SOX2* | 1.33 | 1.46 | n.s. |
| *BEST3* | 1.33 | 1.35 | n.s. |
| *RBP7* | 1.31 | 1.50 | n.s. |
| *BTG2* | -3.63 | -2.03 | < 0.05 |
| *ATF3* | -2.41 | -1.68 | n.s. |
| *ABRA* | -1.99 | -1.46 | < 0.05 |
| *ARG2* | -1.76 | -1.99 | < 0.05 |
| *JUN* | -1.75 | -1.16 | n.s. |
| *ENHO* | -1.69 | -1.49 | < 0.05 |
| *IFRD1* | -1.62 | -1.53 | < 0.05 |
| *PFKFB3* | -1.59 | -1.48 | n.s. |
| *PDZD9* | -1.58 | -1.18 | n.s. |
| *GPA33* | -1.57 | -1.40 | n.s. |

The microarray FC was calculated from the signal log ratios as follows: 2^Signal log ratio^, if signal log ratio ≥ 0 and (-1) x 2^–(Signal log ratio)^, if signal log ratio < 0. Signal log ratios were calculated from *n* = 5 microarrays per group. The qPCR FC was calculated analogously from normalized 2^-ΔCt^ ratios. Normalized 2^-ΔCt^ expression was calculated from *n* = 10 samples per group.

Abbreviations: CON, control group; FC, fold change; IM10, insect meal 10% group.

**Table S8**

Plasma concentrations of carnitine species of pigs fed isonitrogenous diets without (CON) or with 5% insect meal (IM5) or 10% insect meal (IM10) for 4 weeks.

|  | CON | IM5 | IM10 | ANOVA  *P*-value |
| --- | --- | --- | --- | --- |
|  | *µmol/L* | | |  |
| Carnitine | 7.851 ± 0.420 | 8.048 ± 0.936 | 8.494 ± 1.357 | 0.339 |
| Acetylcarnitine | 1.255 ± 0.142 | 1.296 ± 0.136 | 1.390 ± 0.258 | 0.272 |
| Propionylcarnitine | 0.229 ± 0.127 | 0.177 ± 0.045 | 0.208 ± 0.058 | 0.535 |
| Hydroxybutyrylcarnitine | 0.094 ± 0.010^b^ | 0.115 ± 0.015^a^ | 0.109 ± 0.023^a^ | 0.026 |
| Butyrylcarnitine | 0.053 ± 0.006 | 0.057 ± 0.007 | 0.058 ± 0.011 | 0.390 |
| Myristoleylcarnitine | 0.022 ± 0.003 | 0.022 ± 0.002 | 0.023 ± 0.002 | 0.631 |
| Palmitoylcarnitine | 0.017 ± 0.003 | 0.017 ± 0.003 | 0.017 ± 0.002 | 0.854 |

Values are means ± SDs for *n* = 10 pigs per group. Means without a common superscript letter differ, *P* < 0.05.

Abbreviations: CON, control group; IM5, insect meal 5% group; IM10, insect meal 10% group.

**Table S9**

Plasma concentrations of bile acids of pigs fed isonitrogenous diets without (CON) or with 5% insect meal (IM5) or 10% insect meal (IM10) for 4 weeks.

|  | CON | IM5 | IM10 | ANOVA  *P*-value |
| --- | --- | --- | --- | --- |
|  | *µmol/L* | | |  |
| Chenodeoxycholic acid | 4.98 ± 1.67 | 5.26 ± 2.02 | 6.84 ± 2.74 | 0.173 |
| Alpha-Muricholic acid | 0.01 ± 0.01 | 0.01 ± 0.01 | 0.01 ± 0.01 | 0.538 |
| Omega-Muricholic acid | 0.06 ± 0.03 | 0.05 ± 0.03 | 0.08 ± 0.03 | 0.172 |
| Lithocholic acid | 0.20 ± 0.04 | 0.20 ± 0.07 | 0.27 ± 0.13 | 0.172 |
| Hyodeoxycholic acid | 21.0 ± 6.7 | 18.8 ± 8.4 | 25.7 ± 10.6 | 0.217 |
| Ursodeoxycholic acid | 0.43 ± 0.12 | 0.33 ± 0.13 | 0.43 ± 0.18 | 0.212 |
| Glycochenodeoxycholic acid | 6.64 ± 4.90 | 5.14 ± 3.57 | 5.54 ± 3.67 | 0.812 |
| Taurochenodeoxycholic acid | 0.20 ± 0.14 | 0.24 ± 0.11 | 0.20 ± 0.13 | 0.337 |
| Glycolithocholic acid | 0.12 ± 0.07 | 0.09 ± 0.05 | 0.09 ± 0.04 | 0.313 |
| Taurolithocholic acid | 0.01 ± 0.01 | 0.01 ± 0.01 | 0.01 ± 0.01 | 0.235 |

Values are means ± SDs for *n* = 10 pigs per group. Means without a common superscript letter differ, *P* < 0.05.

Abbreviations: CON, control group; IM5, insect meal 5% group; IM10, insect meal 10% group.

**Table S10**

Hepatic mRNA levels of genes involved in fatty acid, cholesterol and bile acid synthesis in the liver of pigs fed isonitrogenous diets without (CON) or with 5% insect meal (IM5) or 10% insect meal (IM10) for 4 weeks.

|  | CON | IM5 | IM10 | ANOVA  *P*-value |
| --- | --- | --- | --- | --- |
|  | *Relative mRNA level (fold of CON)* | | |  |
| *Fatty acid synthesis* |  |  |  |  |
| *ELOVL2* | 1.00 ± 0.52 | 0.87 ± 0.33 | 0.68 ± 0.32 | 0.242 |
| *FADS1* | 1.00 ± 0.34 | 1.12 ± 0.41 | 1.19 ± 0.39 | 0.550 |
| *FASN* | 1.00 ± 0.39 | 0.89 ± 0.29 | 0.82 ± 0.35 | 0.534 |
| *SCD* | 1.00 ± 0.43 | 0.76 ± 0.23 | 0.83 ± 0.24 | 0.295 |
|  |  |  |  |  |
| *Cholesterol synthesis* |  |  |  |  |
| *HMGCR* | 1.00 ± 0.25 | 0.99 ± 0.44 | 1.21 ± 0.50 | 0.422 |
| *LDLR* | 1.00 ± 0.50 | 0.99 ± 0.42 | 0.93 ± 0.29 | 0.942 |
| *MVK* | 1.00 ± 0.23 | 0.85 ± 0.18 | 0.96 ± 0.17 | 0.217 |
| *SQLE* | 1.00 ± 0.29 | 0.76 ± 0.29 | 0.91 ± 0.19 | 0.128 |
|  |  |  |  |  |
| *Bile acid synthesis* |  |  |  |  |
| *CYP7A1* | 1.00 ± 0.30 | 0.86 ± 0.33 | 1.06 ± 0.37 | 0.452 |

Values are means ± SDs for *n* = 10 pigs per group. Means without a common superscript letter differ, *P* < 0.05.

Abbreviations: CON, control group; CYP7A1, cytochrome P450 family 7 subfamily A member 1; ELOVL2, ELOVL fatty acid elongase 2; FADS1, fatty acid desaturase 1; FASN, fatty acid synthase; HMGCR, 3-hydroxy-3-methylglutaryl-CoA reductase; IM5, insect meal 5% group; IM10, insect meal 10% group; LDLR, low density lipoprotein receptor; MVK, mevalonate kinase; SCD, stearoyl-CoA desaturase; SQLE, squalene epoxidase.

**Table S11**

Correlation analysis between significantly altered hepatic phospholipid parameters and significantly altered hepatic mRNA levels in pigs fed isonitrogenous diets without (CON) or with 5% insect meal (IM5) or 10% insect meal (IM10) for 4 weeks.

|  | Correlation coefficient  *r* | *P*-value |
| --- | --- | --- |
| *Hepatic PE P* |  |  |
| *CISH* | 0.182 | 0.337 |
| *HAMP* | 0.187 | 0.32 |
| *ACTG2* | 0.089 | 0.648 |
| *TAGLN* | 0.241 | 0.198 |
| *ADAD1* | 0.077 | 0.672 |
| *GTSF1* | 0.032 | 0.848 |
| *APCS* | 0.000 | 0.988 |
| *DUSP6* | 0.182 | 0.337 |
| *GPRIN3* | 0.000 | 0.915 |
| *SLPI* | 0.239 | 0.202 |
| *CYP26A1* | 0.221 | 0.239 |
| *Hepatic PG* |  |  |
| *CISH* | 0.000 | 0.931 |
| *HAMP* | 0.032 | 0.885 |
| *ACTG2* | 0.055 | 0.782 |
| *TAGLN* | 0.071 | 0.711 |
| *ADAD1* | 0.161 | 0.392 |
| *GTSF1* | 0.032 | 0.853 |
| *APCS* | 0.032 | 0.892 |
| *DUSP6* | 0.247 | 0.19 |
| *GPRIN3* | 0.045 | 0.812 |
| *SLPI* | 0.279 | 0.134 |
| *CYP26A1* | 0.270 | 0.15 |
| *Hepatic PC:PE ratio* |  |  |
| *CISH* | 0.192 | 0.325 |
| *HAMP* | 0.158 | 0.425 |
| *ACTG2* | 0.274 | 0.158 |
| *TAGLN* | 0.349 | 0.069 |
| *ADAD1* | 0.138 | 0.479 |
| *GTSF1* | 0.000 | 0.912 |
| *APCS* | 0.167 | 0.398 |
| *DUSP6* | 0.045 | 0.833 |
| *GPRIN3* | 0.285 | 0.143 |
| *SLPI* | 0.205 | 0.298 |
| *CYP26A1* | 0.063 | 0.737 |
